# Simplification, Innateness, and the Absorption of Meaning from Context: How Novelty Arises from Gradual Network Evolution

**DOI:** 10.1007/s11692-017-9407-x

**Published:** 2017-03-11

**Authors:** Adi Livnat

**Affiliations:** Department of Evolutionary and Environmental Biology and Institute of Evolution, University of Haifa, Mount Carmel, 3498838 Haifa, Israel

**Keywords:** Evolvability, Novelty, Cooption, Parsimony, Gene fusion, Instinct

## Abstract

How does new genetic information arise? Traditional thinking holds that mutation happens by accident and then spreads in the population by either natural selection or random genetic drift. There have been at least two fundamental conceptual problems with imagining an alternative. First, it seemed that the only alternative is a mutation that responds “smartly” to the immediate environment; but in complex multicellulars, it is hard to imagine how this could be implemented. Second, if there were mechanisms of mutation that “knew” what genetic changes would be favored in a given environment, this would have only begged the question of how they acquired that particular knowledge to begin with. This paper offers an alternative that avoids these problems. It holds that mutational mechanisms act on information that is in the genome, based on considerations of simplicity, parsimony, elegance, etc. (which are different than fitness considerations). This simplification process, under the performance pressure exerted by selection, not only leads to the improvement of adaptations but also creates elements that have the capacity to serve in new contexts they were not originally selected for. Novelty, then, arises at the system level from emergent interactions between such elements. Thus, mechanistically driven mutation neither requires Lamarckian transmission nor closes the door on novelty, because the changes it implements interact with one another globally in surprising and beneficial ways. Finally, I argue, for example, that genes used together are fused together; that simplification leads to complexity; and that evolution and learning are conceptually linked.

[C]hange is taking place on many scales at the same time, and ... it is the interaction among phenomena on different scales that must occupy our attention. —Simon A. Levin (1992).

## Introduction

There have been two main ways of thinking about the nature of mutation and how it allows for adaptive evolution. One has been neo-Darwinism. According to it, mutation happens by accident, and natural selection acts as a sieve (though this view started with the mutationists, it lasted to the post-synthesis era; Morgan 1903; Fisher 1930; Dawkins 1986; Beatty 2016). This view not only assumes that mutation is accidental, but often assumes that selection acts on additive (separate) genetic effects, so that it could favor the beneficial mutations and weed out the deleterious ones (Ewens 2004, p. 39; Wade and Goodnight 1998; Provine 1971, p. 165; Provine 1986, p. 241). To this, Sewall Wright added that genetic interactions are also of fundamental importance (Wright 1931, 1932). However, he offered random genetic drift, not a selection-based force, as the cause of the formation of beneficial genetic interactions (Wright 1931, 1932; Provine 1986). These concepts have left traditional evolutionary theory with two important ways of relying on chance: one through accidental mutation and the other through random genetic drift (Kimura 1985).

A substantial addition to the above has been the rise of evo-devo and the related view of evolvability (e.g., Wagner and Altenberg 1996; Hallgrímsson et al. 2005; Müller 2007b, 2008; Brakefield 2006; Hendrikse et al. 2007; Wagner 2014; Laland et al. 2015).^1^ An overarching argument here is that it is not the accidental mutation per se, but its phenotypic consequences, that are subject to selection, and therefore evolution cannot be understood without understanding the developmental process and the genotype-phenotype map, which translate mutation and environmental influence into phenotypic change (Hallgrímsson et al. 2005; Hendrikse et al. 2007). Furthermore, the genotype-phenotype map itself is evolving, and it has been proposed that, from genetic variation, it can generate phenotypic variation that is particularly useful for adaptive evolution (West-Eberhard 2003; Kirschner and Gerhart 2006; Gerhart and Kirschner 2007; Laland et al. 2015).

A different kind of argument, but one that has also been connected to the term “evolvability” (Sterelny 2007; Koonin 2011), is that mechanisms of mutation themselves can evolve under accidental mutation and natural selection (Kimura 1967; Leigh 1970; Ram and Hadany 2014). As a quintessential example, the presumed SOS system in bacteria (Radman 1975) has been interpreted from this direction (e.g., Radman 1999; Caporale 2003; Koonin 2011), implying that bacteria have evolved under repetitive periods of stress to increase their general mutation rate in response to starvation (see Roth et al. 2003; Rosenberg 2001 for reviews and criticisms). Importantly, though, this line of thinking still keeps accidental mutation at the core of the adaptive evolutionary process, first because it relies on accidental mutation and natural selection for the evolution of such mutational mechanisms in the first place, and second because it considers such mechanisms to be additions to the process of accidental mutation and natural selection, a process which still requires accidental mutation at its core (for example, it is presumed that by encouraging many accidental mutations, the bacterial response abovementioned speeds up the search for solutions; e.g., Radman 1999; Caporale 2003; Koonin 2011). Thus, evolvability sensu mutational mechanisms is an addition rather than an alternative to the idea of accidental mutation and natural selection as a sieve.

**Fig. 1:**
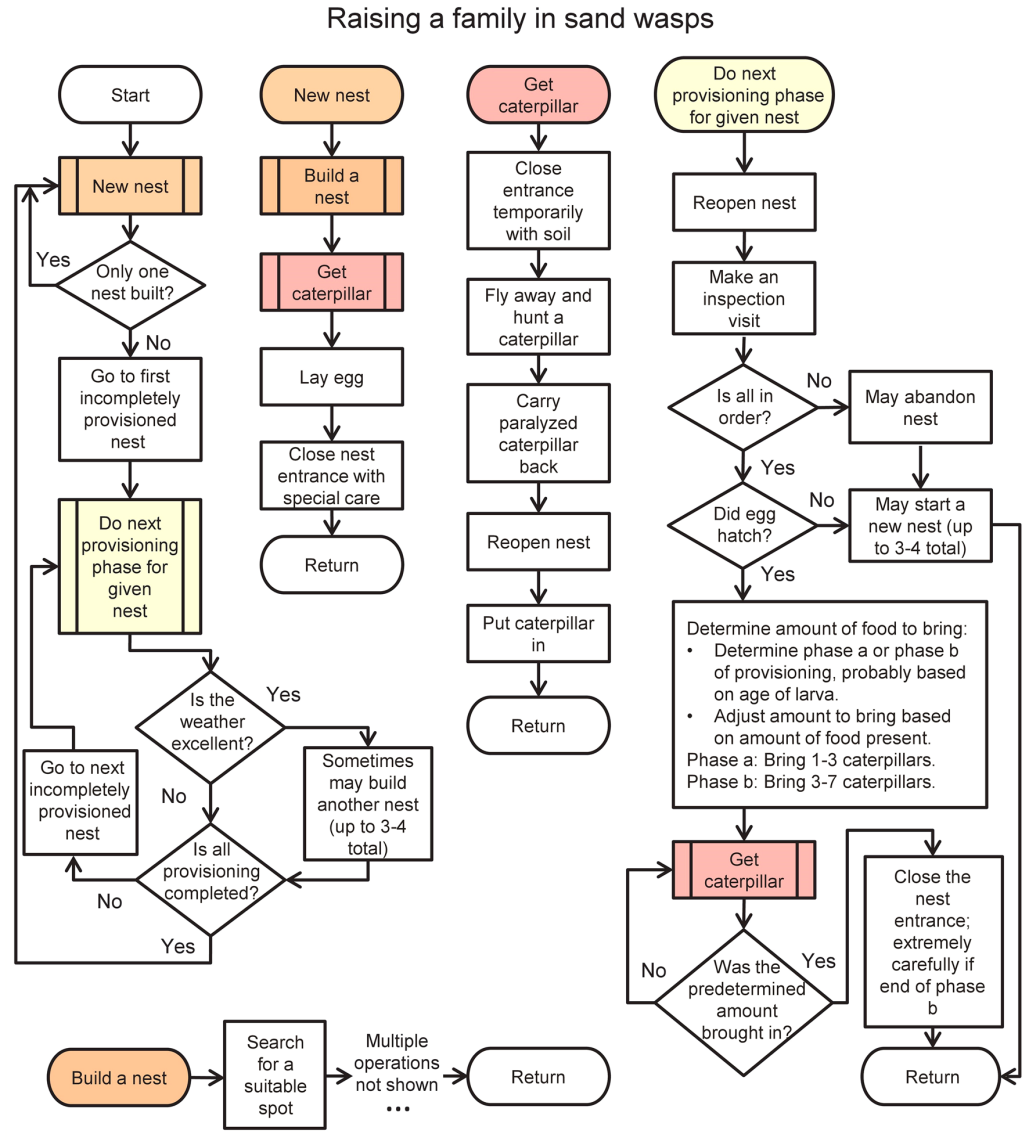
A flowchart describing the algorithmic behavior of nest building, egg laying and provisioning in the sand wasp, *Ammophila adriaansei*. The main procedure begins with “start;” next to it appear the subroutines “New nest,” “Get caterpillar” and “Do next provisioning phase...;” and subroutine calls are denoted by rectangles with double vertical edges. Importantly, the entire apparatus is innate—it is hardwired—and must have evolved somehow. Learning is very limited, and is involved in the acquisition of local orientation but not in the behaviors in the flowchart (Lorenz 1981). For example, removing the larva and food after the wasp has already determined the amount of additional food to bring has no effect on its subsequent behavior, which proceeds like an automaton (Baerends 1941). Interspersed with the activities in the flowchart, the wasp may forage for herself or sleep (not shown). Note that the flowchart is only an approximation, albeit a close one, because of minor incomplete details in Baerends’s (1941) description (such as whether the wasp builds two nests or one after finishing provisioning all existing nests, and several other details)

Coming back to the most basic level of analysis, the other main line of thinking about how adaptive evolution works has been Lamarckism. According to it, the organism can respond through beneficial heritable change to the immediate environment that it senses. This idea relates to modern notions of “directed” or “adaptive” mutation (Luria and Delbrück 1943; Cairns et al. 1988; Hall 1988; Sarkar 1991; Keller 1992; Koonin 2011) as well as notions of Lamarckism through epigenetic inheritance (e.g., Jablonka and Lamb 2005). However, one problem with Lamarckism is that we do not believe, for example, that a hawk could not only sense that it needs sharper vision but also transmit this knowledge down to the level of its genes and then change them in a way that will lead to sharper vision in its offspring at the end of their highly complex course of development. In other words, in multicellulars, Lamarckism represents the unappealing reversal of the apparently one-way function from genotype to phenotype that is development (Koonin 2011) and therefore cannot serve as a general-level explanation for evolution.^2^

Now, there has been a tacit assumption that the two alternatives mentioned above at the most basic level of analysis are the only two alternatives: that either mutation is accidental at the core, or it is Lamarckian, responding in some “smart” way to the immediate environment. One may even ask how there could possibly be an alternative: it seems that either there are mutational mechanisms that are “aware” of the external environment and of genetic changes that would be favorable, or there are not, and then nothing remains to be done but essentially “try changes at random” (or at most add minor modifications such as mentioned above, e.g., adjusting the rate of trying changes at random).

**Fig. 2:**
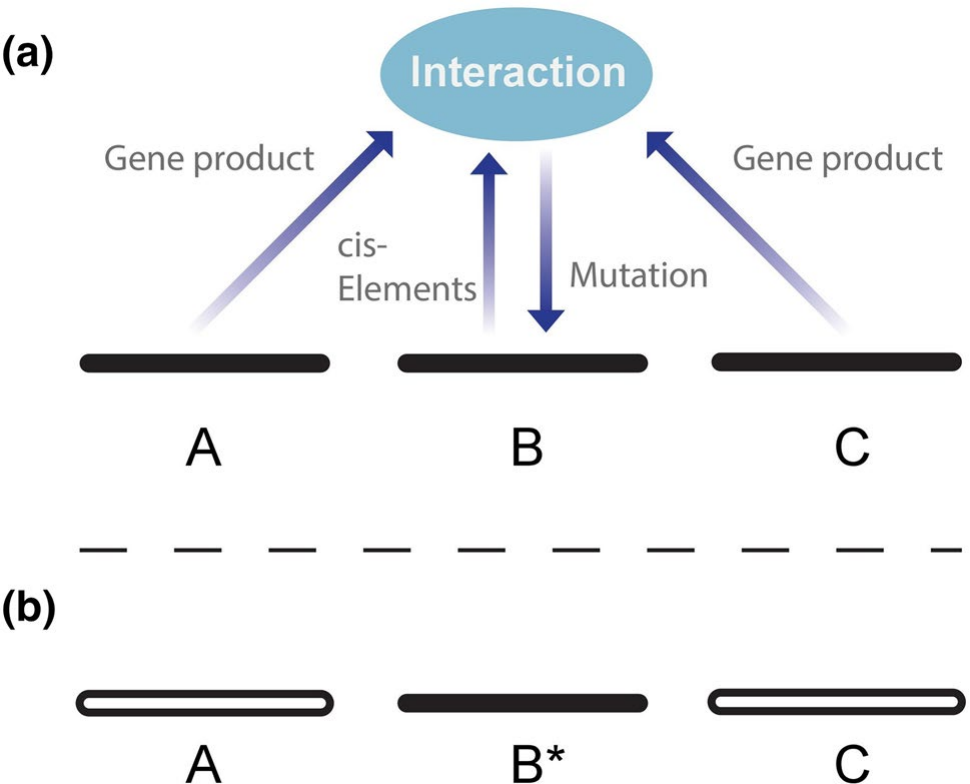
Mutation as a biological process, from Livnat (2013). **a** In this figure we see three loci coming together in a biological interaction through gene products and cis elements. This part of the figure merely represents schematically the gene regulation and interaction that are key to our understanding of molecular and cellular biology. What is new about the figure is that we have not yet fully considered the possibility that there could be a mutation arrow too—that mutation is an outcome of genetic interactions in a heritable mode; i.e., much like genes interact in influencing a classical trait, like the eye or the ear, they also interact in influencing genetic change. Note that the figure purposely leaves open the particulars of the mechanisms involved, as there may be many such mechanisms, and that “mutation” is broadly construed to mean any heritable change. This may involve not only DNA changes but also epigenetic changes. **b** Mutation as an event of information flow and computation changes many things in our conceptualization of evolution. Particularly, the biological process of mutation creates from the combination of interacting alleles across loci a new heritable piece of information—a new mutation—a new allele, B*. Even though the particular combination of interacting alleles will sooner or later disappear due to the sexual shuffling of the genes, information from it can be transmitted to future generations through the mutation. In this manner, the problems of the role of sexual recombination and of the nature of mutation may be tied together

The purpose of this paper is to propose a third alternative. In this third alternative, selection in the sense of differential survival and reproduction is still the force that provides feedback on the fit between the organism and its environment. However, mutation is neither Lamarckian nor accidental. Instead, mutational mechanisms act on information that is in the genome as though by considerations of simplicity, parsimony, elegance, etc. (which are different than fitness considerations). This broadly construed simplification, I argue, operates under the performance pressure exerted by selection—things can only be simplified as long as they keep working—and this simplification under performance pressure is partly responsible for the power of evolution.

Furthermore, I argue that the elements that result from the process of simplification under performance pressure not only perform better but have the capacity to come together in new, useful interactions. In other words, they have the capacity to serve in new contexts they have not been originally selected for. This provides an inherent explanation for cooption in evolution different from treating cooption, like mutation, as a mere accident (Williams 1966).

**Fig. 3:**
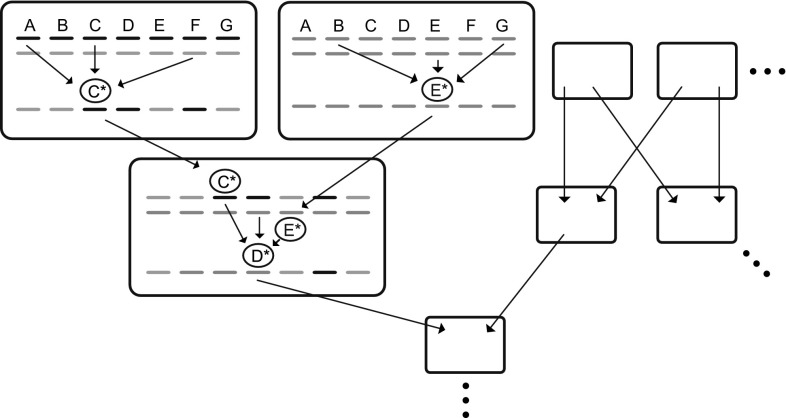
Mutation as an event of information transmission and computation creates a network of information flow through the generations, from Livnat (2013). Each box represents an individual, and in each box, the two sets of lines at the top represent that individual’s diploid genotype (genes A through G), and the set of lines at the bottom represents a haploid genotype transmitted through the gamete. For the sake of demonstration, a small number of mutational events due to interactions between genes is shown in two parents and an offspring (large boxes), although many mutations occur in other genes and in other individuals at the same time. For example, C* represents a mutation in one of the alleles of gene C. Because the *output* of a mutational event in one generation—namely the mutation itself (e.g., C*)—can serve as an *input* into mutational events at later generations (e.g., the event creating D*), non-accidental mutation creates a network of information flow and computation through the generations, from many genes into one and from one gene to many, as well as from many individuals into one and from one individual to many.

In this paper, I will discuss various observations in support of this idea, from gene translocation and fusion (Sect. 2), gene conversion and deletion (Sect. 5.4), and the general notions of error correction and pattern completion (Sect. 5.6) on the molecular level, to the evolution of innateness (Sect. 4), the evolution of stereotypy and ritualization (Sects. 4.8, 4.10), the convergence on an adaptation from a fuzzy and disorganized state to a sharp and clockwork-like state (Sects. 4.12, 4.13), and more on the organismal level. While some of these topics have been the thorn in the side of evolutionary theory since Darwin, here they will be explained from a unifying framework. Why simplification under performance pressure works I will argue by analogy to other creative processes. In model building, in machine learning, and in the very development of science, we know that it is not sufficient for the model or hypothesis to perform—i.e., fit the facts. To prevent over-fitting, the model must also be elegant (Wigner 1960; Hamming 1980). It is a grand fact of nature that the more elegant model, hypothesis, or scientific theory is more likely not only to hold when new information comes to bear on the problem, but also to produce useful predictions that were not necessarily conceived of in advance (Wigner 1960; Hamming 1980). Likewise, I will argue that simplification under performance pressure in biology creates elements that both work well and have the inherent capacity to be coopted (Sects. 3.4, 4.5, 4.8, 4.13, 4.15, 5.7, and throughout).

Crucially, notice that the proposed process of simplification under performance pressure does not lead to ever more simplicity and diminution; on the contrary: It is a well-known fact that duplication of genetic material occurs in various forms and allows the amount of genetic material and genetic information to increase (Ohno 1970; Jacob 1977; Lynch 2007), and simplification operations must be understood within this context. The duplication of elements simplified under performance pressure allows them to serve in new contexts without coming at the expense of the previous function they fulfilled, leading to an increase in complexity. Thus, I argue that, surprisingly, simplification is connected to complexity: under performance pressure, and together with gene duplication upon its various forms, *local simplification leads to a global increase in complexity*.

Notice that, in this proposal, while mutation is not “aware” of the immediate environment and is not Lamarckian, it is non-accidental in a manner not considered before. In fact, it is essential to consider the joint action of this mutation and natural selection.^3^ That is, according to the view proposed here, adaptive evolution is the outcome of two substantial forces that operate together, almost against each other: the pressure of performance and the pressure of simplification.

Notice that this proposed view is substantially different from previous literature. First, although there is no question that evolvability sensu the genotype-phenotype map is of fundamental importance,^4^ it is at first approximation orthogonal to this paper, because this paper is concerned with the antecedent, genetic causes of mutation and how these causes relate to organismal-level evolution.^5^ Second, although this paper will share with evolvability sensu mutational mechanisms the basic recognition that there are evolved influences on mutation, all of its main ideas, including simplification under performance pressure, are distinctly different from those of past literature. Third, although this paper will argue that the causes of the mutations relevant for adaptive evolution under selection are not accidental, as mentioned, it will not invoke Lamarckism.

In addition, this paper will closely connect to past literature on the evolution of novelty (e.g., Müller 1990; Müller and Wagner 1991; Schlichting and Pigliucci 1998; West-Eberhard 2003; Müller and Newman 2005; Moczek 2008; Wagner and Lynch 2010; Hallgrímsson et al. 2012; Hall and Kerney 2012; Brigandt and Love 2012; Peterson and Müller 2013; Wagner 2014). First, it will agree with that literature on the fact that network-level (or system-level) evolution is responsible for the evolution of novelty at both the phenotypic (Müller and Wagner 1991; Hallgrímsson et al. 2005; Müller 2007a; Moczek 2008; Schlosser 2015) and genetic (Wagner and Lynch 2010; Lynch et al. 2011; Emera and Wagner 2012; Lynch et al. 2015) scales and on multiple fundamental points that follow from this (see Sect. 4.16). Second, it will also contribute to that literature in several ways: While past literature on novelty focused on the evolution of morphology, here I will provide complementary examples from the evolution of behavior. In addition, while past literature recognized the centrality of cooption for novelty (Gould and Vrba 1982; Hallgrímsson et al. 2012; Müller 1990; Müller and Wagner 1991; Schlichting and Pigliucci 1998; West-Eberhard 2003; Müller and Newman 2005; Moczek 2008; Peterson and Müller 2013) and the existence of biological phenomena that facilitate cooption, like gene duplication (Müller 2007a; Hall and Kerney 2012) or “weak regulatory linkage” (Kirschner and Gerhart 2006), it has not considered the possibility that the nature of the evolutionary process (the part of it studied prior to the rise of evo-devo) is fundamentally different than that of accidental mutation and selection as a sieve, and that this difference brings out an underlying reason for cooptability, as will be considered here.

This paper is a sequel to a previous paper introducing interaction-based evolution (Livnat 2013), and is a part of that theory. While Livnat (2013) developed the concept of non-accidental mutation by drawing connections between the facts of sexual recombination, the empirical nature of mutation, and more (summarized in Sect. 5.1), the present paper provides a way of understanding the big picture of how natural selection and non-accidental mutation work together. It relies on non-accidental mutation to drive simplification.

The paper will be structured as follows. The next section will offer a network-based view of evolutionary change. Section 3 will introduce the idea of simplification under performance pressure. Together, these two sections will propose how non-accidental mutations can be useful for evolution yet not be Lamarckian. Building on these new concepts, Sect. 3 will offer new insights on the emergence of novelty in evolution. Section 4 will bring a large number of empirical observations in support of the view proposed here, focusing on such phenomena as the evolution of innateness, stereotypy, and ritualization. Insodoing, it will argue that *automatization is at the essence of the evolutionary process.* Of particular importance will be Sect. 4.13, where all the concepts developed up to that point will come together in one empirical example with an emphasis on the evolution of novelty—that of the evolution of egg retrieval by backward walking in birds. Finally, Sect. 5 will revisit the molecular level in light of the concepts developed, discuss the nature of mutational mechanisms and draw a connection between evolution and learning, including machine learning, underscoring the importance of the algorithmic lens (Papadimitriou 2007; Karp 2011) for our understanding of evolution.

## A Contextual View of Genetics

A single example of non-accidental mutation will help to fix ideas. Consider the genes encoding cyclophilin A (CypA) and TRIM5. CypA is a highly abundant cytosolic protein (Haendler and Hofer 1990) that, among its various activities, potently binds several retroviral capsids, including HIV-1 (Kaessmann et al. 2009). TRIM5 is a restriction factor that recognizes and inactivates incoming retroviral capsids (Virgen et al. 2008). Interestingly, a copy of the CypA gene has retroposed into the TRIM5 gene independently in at least two different simian lineages (Virgen et al. 2008; Nisole et al. 2004; Sayah et al. 2004; Liao et al. 2007; Brennan et al. 2008; Wilson et al. 2008; Newman et al. 2008), and the resulting TRIM5-CypA fusion protein appears to provide strong protection against certain lentiviruses (Nisole et al. 2004; Sayah et al. 2004). Not only have these two genes fused at least twice, there are many other TRIM genes, and tests of artificial fusions of the CypA domain to some TRIM motifs have shown that they too can provide retrovirus protection (Zhang et al. 2006; Yap et al. 2006, 2007), yet *TRIM5* specifically repeats in both fusions mentioned above (Virgen et al. 2008).

Here I argue that this gene fusion has not been accidental, but has been the result of mutational mechanisms (see also Livnat and Papadimitriou 2016a). In particular, I argue the following. Genes that are used together are more likely to be transcribed at the same time and even in the same place in the nucleus (e.g., in transcription factories, which bring remote loci together; Jackson et al. 1993; Edelman and Fraser 2012; Papantonis and Cook 2013). Furthermore, since genetic information indicating that they work together (like shared cis elements and transcription factors that bind to them) is present in the DNA and accessible in the germline, they can be transcribed together in the germline due to the latter’s transcriptional promiscuity (Kleene 2005; Livnat 2013) even if they work together specifically in the soma. Hence the two loci can be brought close together and be open at the same time in the germline. This will make them incomparably more likely than other genes to be fused together by reverse transcription, which we know to occur in the germline (Brosius and Tiedge 1996; Brosius 1999), or by other mechanisms (e.g., recombination-based ones). Furthermore, and interestingly, the fact that there is transcriptional promiscuity in the germline allows many somatic as well as germline genes to participate in such operations. In general, therefore, I argue that copies of genes that are used together repeatedly and persistently in a certain context are more likely to be genetically fused; or in short: *genes that are used together are fused together* (see also Livnat and Papadimitriou 2016a).

When considering translocation and cooption it has commonly been assumed that sequences just jump one day by chance from one locus to another, and, on rare occasions, fortuitously acquire a new use there. Indeed, because genes are either fused or not, it may even appear at first sight as though molecular cooption could not be an outcome of a gradual evolutionary process, fitting with the view of mutation as an unexpected, stochastic event. However, I argue here that gene usage and genetic change are mechanistically connected, and that, more specifically, gene fusion is preceded by gene action: First, a gradual evolutionary process leads a pair of genes to interact tightly in a certain context. Then, their fusion does not require a unique accidental event of low probability but rather is facilitated by the genetic mechanisms mentioned above. In fact, the same fusion can then happen in different individuals independently, which helps to explain both recurrent gene fusion in genetic disease (Li et al. 2008; Osborne 2014) and parallel gene fusion in evolution (for a related discussion, see Carvalho et al. 2010; Livnat 2013). It even raises the intriguing theoretical possibility of a sharp evolutionary transition within a species from a pre- to a post-fusion state without a selective advantage.

Are genetic evolutionary mechanisms such as the mechanistic gene fusion above important for our understanding of the essence of the evolutionary process, though? One way to demonstrate their potential importance is through the connection between the above mechanistic gene fusion and Hebbian learning (Stephen Pacala, personal communications). According to Hebbian learning, when one neuron participates in firing another neuron repeatedly and persistently, the strength of the synapse between these two neurons is increased (Hebb 1949). Thus, it has been said that neurons that fire together wire together (Löwel and Singer 1992). Hebbian learning is important because it is one of perhaps many operations of network change that allow the brain to learn in the context of continual input from the outside. In the above, we have a genetic evolutionary operation that is Hebbian learning–like (Livnat and Papadimitriou 2016a): genes used together are fused together. Much like local operations of network change are believed to be essential for learning in the context of continual input, this connection opens our eyes to the possibility that mechanisms of genetic change, in the context of natural selection, are fundamental to evolution.^6^

Importantly, notice that, like Hebbian learning, the genetic fusion mechanism above *exemplifies simplification:* genes that used to be regulated by two separate lines of regulation are now regulated as one (see more in Sect. 2.2). In addition, we see here an example of the evolution of innateness at the molecular level: what was previously a phenotype—an interaction between two genes (Livnat and Papadimitriou 2016a)—is now a ready-made gene. Simplification and innateness are two main themes that will be discussed in this paper.

### Cooption at the Phenotypic Level is Due to a Gradual Process

Let us now consider an example from the organismal level, which shows that simplification, cooption and innateness occur there as well. This will be the example of the inciting ceremony in ducks (Lorenz 1941/1971, 1958, 1966). In the European common shelduck (*Tadorna tadorna*), when the female is standing near her mate, her aggression instinct is triggered by the presence of neighbors, and she may run toward them with her neck stretched, which is the threat posture in ducks (Lorenz 1966). As she approaches them she naturally becomes fearful, turns around and flees back toward her drake.^7^ Approaching her drake, the former instinct is triggered again. In those cases where her breast is still facing him, she turns her neck back to threaten the neighbors over her shoulder. This behavior by the female can incite her mate to attack the neighbors. Note that the angle between the neck and the body of the female is entirely dependent here on the situation—her body orientation is due to the location of the drake and her neck orientation is due to the location of the neighbors (Lorenz 1966).

Lorenz followed the homologues of this behavior in other duck species and suggested that the to-and-fro movement such as seen in the common shelduck has gradually become ritualized, so that, in the ritualized forms, the female does not perform the to-and-fro but stands near her drake; and, most interestingly, the two elements of orienting the body toward the male and stretching the neck over the shoulder toward the neighbors—which in the non-ritualized forms are triggered separately by the environment—have become fused together (Lorenz 1941/1971, 1958, 1966). For example, in the East European-Asiatic ruddy sheldrake (*Tadorna ferruginea*), the neck and body orientations are still controlled separately, but in most of the cases the female stands with her breast to the drake and her neck pointing backwards (and very rarely this behavior may be performed without a neighbor present) (Lorenz 1966). And in the mallard (*Anas platyrhynchos*), the same breast-to-the-male-and-pointing-backwards is observed, but now this posture is compulsory and, at high excitation, which activates the instinct (the same relationship between excitation and activation of instinct exists for many other instincts), the female is compelled to turn her neck over her shoulder even if that means that the neck moves away from the neighbor (Lorenz 1958). Thus, two elements of behavior, previously triggered separately by two separate environmental triggers, have become fused together and triggered as one. Finally, in the golden-eye (*Bucephala*), where the movement is highly ritualized (see below), the presence of a conspecific is not even required (Lorenz 1966).

Interestingly, along with the evolutionary change of form of the behavior, there has been also an evolutionary change of meaning. In the species with the less-ritualized form, the behavior has the effect of inciting and is related to territorial behavior. However, note that it already has in it an element of pair-bonding, or team work. In the more ritualized cases, this pair-bonding meaning has moved to the fore: in the mallard, though it sometimes still elicits a demonstration of attack by the male, inciting serves mostly as an invitation to pair-bond; and in the golden-eye, the inciting has become almost entirely independent of the presence of neighbors, and takes a highly ritualized, exaggerated and rhythmic form of neck movements over one shoulder and then over the other (and rhythmic movement is indicative of highly ritualized behaviors in general).

It is due to the highly surprising nature of this example and others that Lorenz has been accused of Lamarckian thinking. However, many examples of this sort exist, and I will argue that they are explained not by Lamarckism but by network-level evolution (Sects. 2.2, 4).

What is important to notice in the two examples discussed so far is as follows. In both of them, we see a *gradual process* arising from *preexisting interactions*. A novel phenotype (the fused protein in one case, the ritualized display in the other) arises from the change in context in which preexisting elements (preexisting genes, movements) are embedded. In fact, what was once an interaction has now become an object: in the case of *TRIM5-CypA*, a hypothesized interaction between two separate genes is succeeded by a gene fusion; and in the evolution of the inciting ceremony, two separate behavioral responses to two separate environmental triggers (orienting the body toward the drake and threatening the neighbors over the shoulder) has now become fused into a new instinct. These cases show that the source of novelty can be in system-level changes. In both cases, novelty arises *not* from a point-wise change, *not* suddenly and *not* without relation to preexisting heritable information.

Among other things, we also see local simplification in both cases: in simians, what previously required the separate transcription of two genes now requires the transcription of one, and in ducks, a roundabout to-and-fro behavior has now turned into a stationary clear display. These aspects and more will be explored in-depth in this paper with the use of various examples and by developing concepts.

### Network Evolution and Its Operators

I will now propose a verbal model that ties shifts in context to network-level evolution. The model is purposely described at a high level because its role is to elucidate concepts, not to provide mechanistic detail.

Consider that in the course of genetic evolution, the network of genetic interactions gradually changes as a whole. Many changes take place across the genome and across time, and these changes interact. This process involves regulatory changes that can rewire the genetic network (Carroll 2005), such as movements of transposable elements carrying with them cryptic enhancer/promoter sites and multiple mutations activating those sites (Lynch et al. 2011). Even a regulatory change that at first sight appears only to change the strength of an existing connection between two nodes—e.g., to increase the effect of a regulator on its target—can effectively cause rewiring, because there is no sharp boundary between the case where the regulator has a negligible effect on its target (in which case the two nodes can be said to be effectively disconnected) and the case where it has a non-negligible effect (where the two nodes can be considered to be connected).

Rewiring means that, in the course of evolution, the connections between some nodes in the network become tighter and the connections between other nodes become weaker, and recognizing it is important. When the connections between nodes become tighter, they come to be regulated more and more as one unit, and a new module arises. What in the beginning may have been two separate elements regulated by two separate lines of control can gradually come under one line of control. As will be understood later, this change represents the arrival of a new automatic unit. Furthermore, when this coming together of genes is preceded by the duplication of those genes and their regulatory elements, this new module does not arise at the expense of previous ones, but represents a total increase in the number of modules; and together with this increase in the number of modules comes an *increase in the extent of higher-level interactions* between modules (since all the modules must ultimately come together into one organism, and now there are more of them^8^).

While the term “module” usually refers to a set of tightly interacting genes, a rather basic module or unit is an exon; and because exons in separate loci may interact through trans-splicing, or through protein-protein interactions, etc., the same kind of process of tightening and weakening connections between nodes can cause the coming together of two previously interacting exons into a gene, or gene fusion. Such a fusion may be long in the making. This shows us a case where a new elementary unit evolves from an interaction—from a process—and where a process becomes an object—a gene.^9^ And as an object, it begins to accept the kind of operations that the system can apply to other objects. It is now interacting directly and indirectly with many other units.

A critical point in the above now calls for reflection. It takes time for two elements to undergo separate regulation and transcription in order to come together into a functional unit or interaction. But when they come together evolutionarily into one genetic unit, regulated as one and performing through one product, this time is cut to zero. Previously, the joint effect of these two elements came into being as developmental interactions do; now it is “innate”—it is a gene. It no longer needs to be constructed from more-elementary units, and it exerts its effect in interaction with other (now-peer) elementary units, in which context it has phenotypic meaning. The emphasis here is not on the actual amount of time cut, but on the local network simplification that this process represents.

Thus, in the gradual fusion of two elements into one, we see a sense of evolutionary acceleration of developmental interactions; and if this fusion is preceded by the copying of those two elements, we see at the same time an increase in the “genetic vocabulary,” which comes together with an increase in the extent of higher-level interactions—an increase in complexity.

Having thus formed a clear view of acceleration and the arising of new interactions with the help of the gene fusion case, it is important to step back again and observe these two aspects from a broader viewpoint. It is enough to consider the copying of modules and the changing of regulatory connections between them (prior to considering actual gene fusion) in order to notice that these changes of connections can be seen from two angles: When we look at the lower levels of organization—at the tightening of connections between nodes—we see an increase in innate abilities. When we look at the higher levels of organization—at the increase in the extent of interactions between modules due to the appearance of new modules—we see an increase in the complexity of the life-form, the phenotype. Importantly, these are two facets of one integrated process: the new parts observed at the lower levels (which are due to constriction) and the new whole (which is due to the increase in the extent of high-level interactions) *coevolve*. The novelty comes from network-level change, not from a sequence of independent, atomistic changes. And, as will be discussed, adaptation comes together with innateness—with automatization.

Notice also that there are useful *operators* in the evolution of networks: The *copying* of nodes along with their connections adds syntactic material to the network from the inside, which serves as a basis for increasing complexity. The *chunking* of nodes and the *severing of connections* between nodes allows nodes to separate from their previous context and join new contexts gradually.

One important point about this section is that there is a sense of an Archimedes screw–like operation in network-level evolution. An Archimedes screw is a helical surface wrapped around a shaft inside a pipe that is designed to carry water up from one side of the pipe to another as the screw rotates. Each point rotates at its own level, yet due to that rotation, water flows up. Likewise, in network-level evolution, when a genetic interaction is replaced by a gene in the course of evolution, or when a behavioral sequence with environmental triggers is replaced by an instinct, there is a sense of a transfer of meaning from higher to lower levels of organization—from phenotype to genotype—despite the fact that materialistic changes like movements of genes are confined to their respective levels; that is, the phenotype does not actually become a genotype. This will help us replace the notion of novelty from a local genetic accident with the following: novelty arises at the system level and is then crystallized in an evolutionary process based on mutational operators working under natural selection.

### The Gradual Evolution of Innateness of Alternative Splicing Patterns Results in Exon Shuffling

As mentioned in Sect. 2, traditional discussions on the evolution of chimeric genes seem to assume that they arise by sudden, fortuitous events. In contrast, I argued that they are generated by gradual evolutionary processes that set the conditions for fusion. Importantly, the same kind of mechanisms discussed in the case of the *TRIM5-CypA* fusion, under the network-based view of evolution proposed in the last section, can be connected to the general question of how exon shuffling and alternative splicing patterns evolve.

“Exon shuffling” refers to the fact that homologous exons can appear in different genetic contexts in different species or even the same species. “Alternative splicing” refers to the fact that, in eukaryotes, multiple products can be generated from different combinations of exons (whether the exons are taken from nearby as in the case of cis-splicing, or from different loci as in the case of trans-splicing). The former implies a process in evolutionary time. The latter is a process in developmental time. Now, we know that there are cases where the same exons are being trans-spliced in one species or strain but cis-spliced in another (Kong et al. 2015), such as the exons of the separate *eri-6* and *eri-7* in *C. elegans* strain N2 and their fused homologs in *C. briggsae* and in other strains of *C. elegans* (Fischer et al. 2008). Likewise, we know that some functions are achieved by multiple single-module proteins in one species but by a single, multi-module protein in another, where the genetic sequences encoding these modules are fused (Graur and Li 2000). For example, the activities required for the synthesis of fatty acids from acetyl-CoA are carried on by discrete monofunctional proteins in most bacteria, and are encoded by two unlinked genes in fungi (Chirala et al. 1987; Mohamed et al. 1988) and by a single multi-exon gene in animals (Amy et al. 1992) (see Graur and Li 2000). While a connection between exon shuffling and alternative splicing was suggested as soon as the latter was discovered (Gilbert 1978), I offer to sharpen the nature of this connection, since the abovementioned facts connect with the preceding sections: When one of two exons that used to be trans-spliced is now translocated and fused to the other genetically (or becomes cis-spliced to it), we have a case that fits the *TRIM5-CypA* fusion case—that could be explained mechanistically in the same (or a similar) way. In other words, translocations that are part of the evolution of alternative splicing patterns that would have been considered from a traditional perspective as sudden, stochastic events could be outcomes of gradual evolutionary processes involving genetic mechanisms in the sense of Sect. 2. Thus, I argue that exon shuffling is the *gradually evolved, innate state* of alternative splicing. Namely, what is constructed in developmental time is *gradually* replaced in evolutionary time with new innate elements and a new developmental construction. Specifically, when two exons previously spliced together at the RNA level are now fused at the DNA level, it is a case where a process in developmental time—a splicing pattern affected by various factors—has become an innate object—a gene fusion, emancipated from the influence of those factors.

The gradual evolutionary process that brings two genetic elements to work tightly together and sets the genetic conditions for mechanistic fusion can itself occur according to the principles of interaction-based evolution as discussed by Livnat (2013). One may hypothesize that alleles evolving at multiple loci gradually change the regulation of the alternative splicing pattern in the focal gene as well as in other, coevolving genes. Genetic information from these loci can then be gradually collected by non-random mutation (Livnat 2013), setting the new genetic sequences as well as the new alternative splicing patterns that we see today. In other words, many mutation-writing events, in each of many individuals, in each of many generations, under natural selection, gradually pave the way for network evolution at the gene level, as will be discussed in Sect. 5.

Consistent with the above, Stone and Schwartz (1990) hypothesized that separate genes whose products first aggregated in the cytosol to form a functioning enzyme could later become fused at the DNA level. They suggested, as an example, that the different lobes of an enzyme such as glyceraldehyde-3-phosphate dehydrogenase may have come from separate genes far in the past, before those genes became genetically fused; and that this could also explain the existence of a family of dehydrogenases, each of which has fused the same gene encoding the NAD binding protein with differently mutated copies of the gene encoding the substrate binding domain. Furthermore, West-Eberhard (2003) predicted that the connection between evolution and development will be found in the connection between exon shuffling and alternative splicing and in other phenomena (West-Eberhard 2003). This paper agrees with them on these points and adds that the gene-fusion case is merely an example of a more general principle according to which elements absorb novel phenotypic meaning from their gradually changing context under interaction-based evolution (as will be discussed more in this paper; see also Livnat 2013).

### Novelty Comes Neither from a Point Nor from DNA “Misspelling”

Now, gene fusion may be discussed as one topic, and cooption as another. But they are actually two sides of the same coin. In both cases we see elements or copies thereof leaving their previous context and moving to a new context. But although fusion and cooption are parts of the same process, the case of fusion is especially grabbing to the eye, because it shows the creation of a new elementary unit in a manner that traditional theory has not prepared us for. In traditional theory, there is point mutation and presumed genetic novelty from it,^10^ and there is gene duplication followed by point mutations in the duplicates (Lynch 2007); but there is no evolutionary process where *a process can become an object*—where a new elementary unit is created from something that previously was an interaction (indeed, this new elementary unit absorbs new meaning from its gradually changing context).

Two remarks are important. First, this manner of creating a new elementary unit requires the existence of a hierarchical structure of organization—a network—where, by a gradual change in the network, such a process can happen. Since this hierarchical structure does exist and is a fundamental aspect of nature, it is an advantage of the present theory that it engages this structure. Second, the gradual creation of a new elementary unit from what was previously an interaction is important because it shows us that the *barrier between “unit” and “interaction” has been broken*. There is no sharp dividing line between elementary units and higher-level interactions. The collapse of the gene concept as a well-defined unit (Gerstein et al. 2007) is supportive of this absence of a sharp division between process and object^11^ and fits with a *gradual* process of gene formation (Livnat 2013; Carvunis et al. 2012).

Indeed, while this paper is consistent with modern literature on novelty in important ways to be discussed in Sect. 4.16, it is fundamentally different from the traditional view of evolution prevalent at the foundation of the modern synthesis. Not only does the traditional view focus on object minus context and imply that novelty arises in the object by a local genetic accident that emanates this novelty “upward” to the complex system—novelty from a point—but in addition, this point-like change is considered to be an error akin to a “misspelling.” If we let genes be words, metaphorically speaking, and let the phenotype be the technology that they describe, then the traditional notion of mutation can be exemplified by misspelling unintentionally the word “incubate” while making all effort to copy it accurately, and thus suddenly getting the idea of inventing an incubator. Whereas in reality, the incubator (i.e., technology, or the phenotype) is invented by the use of many concepts described by putting together many words; and in the long-term, the whole complex object that is an incubator might even be given a standard, symbolic name by which this whole has come to be referred to conveniently: the word “incubator,” generated by a standard operation of adding the appropriate suffix to a preexisting, useful word. From the view of interaction-based evolution, to say that the “misspelling” of genes (accidental mutation) is the source of biological novelty is to make a mistake in understanding the nature and the role of the bottom level of the genetic interaction hierarchy, similar to saying that the misspelling of words creates technology.

Considering all of the above, a main point of this paper is that evolution is a “bottomless system”.^12^ One cannot define all words in the dictionary in terms of other words without getting into a circularity. Ultimately, the meaning of words comes from the context of their usage; that is how language is learned and even how it evolves. The genes are similar in this regard. Their meaning comes from the context of their usage. They themselves are nodes in a network, in development as well as in evolution. Thus, the bottom of the hierarchy of biological interactions—the genetic sequence—is not a stable ground upward from which life is built. Mutation is not a local accident that brings innovation all on its own as though there is no living network that it needs to connect to. The process of genetic change is a complex one where the connections between nodes in the network become stronger and weaker as they form modules that *absorb meaning from context*.

Traditionally, we have been thinking about an accident, disconnected from the living network, as an event that creates new genetic information. This was conceived as a point-like event, which then emanates the novelty that it brings about to the phenotypic level (but see Sect. 4.16 for modern developments). I argue instead that novelty arises from network-level change, and that this requires non-accidental mutation that executes network change in a syntactic and evolving fashion (Livnat 2013).

## Simplification and Novelty

For Darwin as well as for Fisher (Fisher 1930), complexity evolved in cases where an increase in it was needed for an increase in fitness. However, the question of why complexity evolves has never been resolved (Wagner 2014, p. 11). I argue here that simplification under performance pressure leads to both complexity and novelty.

This section will be entirely devoted to discussing concepts. Once they are discussed, numerous empirical examples will be brought forth in Sect. 4.

### Simplification Under Performance Pressure Leads to Complexity

Several points in the present theory may be organized under the heading of “simplification,” each of which comes with its own corresponding increase in complexity, as shown below.

#### *Modularity and Simplification*

- Simplification and modularity are tightly connected concepts. A module serves multiple contexts—in fact it is defined by them—and in the case where one serves the many, as in the case where one explains the many, there is frugality, parsimony, or simplification.
- I discussed above the gradual appearance of modules in networks. A key example of the appearance of a module was the fusion of two genetic elements. Here, the developmental process originally putting them together is *simplified away* in the course of evolution. More generally, the gradual arising of new modules from a previously complex, interconnected mass of nodes is the evolutionary streamlining, or simplification, of development. Elements inside a module are emancipated from the complex influence of elements that are now outside of it and are no longer connected to it.

#### *Innateness and Simplification*

- As will be shown soon, an extension of the last point is the evolution of innateness, which involves evolved independence from environmental triggers. During evolution, an evolving trait can become emancipated from complex environmental influence involved in the development of an adaptive ancestral phenotype as a more orderly, simplified and compartmentalized developmental process evolves. Thus, the evolution of innateness involves simplification: what consumed developmental (and sometimes learning) time is simplified away in the course of evolution.

Now, the cases of simplification described above come together with an increase in complexity. As argued earlier, due to the duplication of genes, the formation of a new module need not come at the expense of old modules. The increase in the number of modules or elementary units comes together with an increase in the number of interactions between such modules or units, which represents an increase in complexity. Simplification is what we see when we look at the modularization of an interconnected mass, and complexity is what we see when we look at emerging interactions involving newly formed modules. *Local simplification leads to a global increase in complexity.*

#### *The Final Touch of Perfection*

There are observations that show the development of organs or tissues taking ever straighter paths over evolutionary time (Müller 1869). For example, in cetacean embryos (e.g., whales and dolphins), hind limb buds still appear fleetingly in development and grow to a small size before they are removed (Sedmera et al. 1997). In such cases, it is evident that, over evolutionary time, the developmental process gradually comes to spend less and less time and energy on developing structure that is slated to be superseded by another or to be removed later in development.

How does it happen that evolution straightens up developmental paths? A neo-Darwinian answer is that the savings of time and energy are directly favored by natural selection, so that a whale that acquires by chance a mutation that reduces the development of the useless bones by even a small amount gains a slight benefit in terms of survival and reproduction, and thus accidental mutations of this sort are passed on preferentially. However, is it reasonable that a slight straightening of the developmental path of useless, internal small bones is truly enough to make such an impact on differential survival and reproduction that would be noticeable, when many and more important other individual differences may be contributing to differential success?

This problem of the obliteration of rudimentary organs is a very old one (Darwin 1859). Darwin himself *agreed* that it was unreasonable to explain the removal of rudimentary organs as an outcome of natural selection based on minute economic considerations alone (Darwin 1876, pp. 309–402). Indeed, he held steadfastly to the Lamarckian “laws of use and disuse” and more to explain them, which is remarkable in light of the sections to follow on innateness. The same problem was discussed by Weismann hand in hand with that of the final touch of perfection—how adaptations become perfected beyond what may be possible by traditional means (Weismann 1902). Wiesmann, who is considered the father of neo-Darwinism, explained the final touch of perfection by suggesting a principle of “momentum” or “inertia,” where a mutation in a certain direction will be followed by others in the same direction, so that noticeable, selected improvements of economy will be followed up by minute, unselected ones (Weismann 1902). Interestingly, this point is completely outside of the view based on random mutation and natural selection, a view which traces its ideological origins to Weismann (Winther 2001; Gould 2002). Thus, it is instructive that the two greatest pillars of neo-Darwinism went to great lengths to look for alternatives to the twentieth century’s explanation-of-choice based on economical considerations alone.^13^

Interaction-based evolution offers such an alternative: simplification under performance pressure. Instead of relying on economical considerations under accidental mutation and natural selection alone, it argues that an active, mechanistic force of simplification drives the “final touch of perfection,” both in the complete obliteration of a trait and in the crystallization of adaptation, as will be discussed in Sects. 4.8, 4.12 and 4.13 (notice, for example, the prediction that stereotypy in general, as well as schematization in rituals, is due to simplification under performance pressure rather than due to benefits of improved clarity of signals; Sect. 4.8).

Now, notice again the connection between simplification and complexity: the intriguing straightening of developmental paths demonstrated by the unexplained old observations is tied to the “final touch of perfection”—a convergence on an optimum in the evolution of a complex adaptation.

#### *Convergence, Simplification and Complexity*

According to interaction-based evolution (Livnat 2013), sexual reproduction and non-accidental mutation combine information from different loci and from different individuals that succeeded in survival and reproduction. Alleles at different loci concomitantly spreading in the population do not each bring an independent piece of the phenotype to all individuals, but rather interact with each other. Thus, an adaptation evolves at the level of the population as a whole, at the same time as it becomes more genetically stable (see Sect. 4.10; Livnat 2013). This process slowly *gives rise to the true, common reason for success shared by individuals*, as the initially many and highly variable ways by which different individuals approximate the adaptation only roughly at first are gradually superseded by an adaptation, uniform across individuals (see nest-digging by sand wasps, discussed in Sect. 4.12; see also Livnat 2013). We may now note that in this replacement of many by one—of the different rough approximations by one uniform adaptation—there is simplification. At the same time, this one that replaces the many is a complex adaptation—a point of optimality. Therefore, simplification and complexity again come together: the complexity that is in the different ways of approaching an adaptation has been converted into the complexity of the adaptation itself.

#### *Simplification and Complexity: Summary*

We have seen that each of the above connections to simplification comes together with an increase in complexity. Could simplification under performance pressure (e.g., under selection) be the cause of the evolution of complexity? This question is best answered together with another, related question, discussed next: What is the source of novelty in evolution?

### The Problem of Novelty

This paper will be entirely consistent with foundational literature on the origin of novelty (e.g., Müller 1990; Müller and Wagner 1991; West-Eberhard 2003; Müller and Newman 2005; Moczek 2008; Wagner and Lynch 2010; Hallgrímsson et al. 2012; Hall and Kerney 2012; Brigandt and Love 2012; Peterson and Müller 2013; Wagner 2014) in that both agree on the fact that network-level evolution is key to novelty and on multiple fundamental points that follow from it (Sect. 4.16). It will also attempt to contribute to that literature in several ways. However, these connections will only be clear in Sect. 4.16, after the required concepts and examples are covered. In the meantime, suffice it to say that I will use the term “novelty” broadly in a way that is different from how it has been used before to simply mean “something that is new” while keeping an eye on the fact that any heritable change, whether big or small, represents some amount of new genetic information (it requires some genetic or epigenetic change/s). The question I will focus on is where does this new genetic information come from? Does it come from a local genetic accident and the only “smarts” involved is in the genotype-phenotype map? I will connect these questions to simplification under performance pressure and to network-level evolution. My high-level point will be that, even if genetic changes are mechanistically driven, novelty can still arise from the unexpected interaction between these changes. Thus, simplification under performance pressure and network-level evolution remove the need for accidental mutation to be the source of new genetic information (Sect. 5.2).

### A Dilemma Regarding Novelty and the Nature of Mutation

Besides the fact that it does not apply to multicellulars in an appealing manner, Lamarckism has another problem which is fundamentally related to novelty: hypothetically speaking, even if mutational mechanisms knew what would have been favored by natural selection in a particular organism at a particular point in time and how to produce it, this would not have solved the problem of how novelty in the sense above arises, because the novelty would have been in how such supposed mechanisms acquired that particular knowledge to begin with. Indeed, it is easy to erroneously think that, if there is knowledge of the thing to be produced, there is no novelty, and if it is to be produced without knowledge, it must be produced by accident. Thus, we can understand the immense attraction of accidental mutation from a traditional perspective: First, it requires no Lamarckian mechanism transferring knowledge from the macroscale to the genotype. Second, accident has no preconceptions, and it seems to have been believed that it can invent the required new genetic information.

As articulated by Livnat (2013), it is a key property of interaction-based evolution that the non-accidental mutation that it proposes does not circumvent, but rather works together with, natural selection, and is strictly non-Lamarckian. This removes the first reason above to hold on to accidental mutation. However, the second problem still needs to be addressed: if the mutational mechanisms are not “aware” of the environment, how could mutation be anything other than accidental? How could the ultimate source of new genetic information in evolution be anything other than random mutation?

### Simplification Under Performance Pressure Leads to Novelty

To try to answer this question, let us allow ourselves to step outside of evolution and look at how novelty arises in other creative processes.

Consider the development of scientific theories. It has two fundamental principles. First, theories need to fit the data—they need to *perform*. Second, they must be parsimonious. When we take disconnected facts and find a theory that explains them all in one, we create a more parsimonious picture of reality than existed before. It is a fortunate fact of nature that when we do so we often obtain a model of reality that will hold better when new and unexpected data later arises and that will lead to findings not previously expected.

A well known example of the use of parsimony in science is the Copernican revolution—the placing of the sun instead of the earth at the center of the solar system. Copernicus proposed this model not because it allowed him to make better predictions of the movements of the planets, but because it was simpler on an essential point (Singh 2004). This simpler model paved the way to future science, generally fitting with major later findings by Kepler and Galileo, like the phases of Venus.

From this and many other examples we see that the pursuit of parsimony does not merely provide elegance per se. Parsimony *expectedly brings the unexpected*—useful things that were not initially predicted and were not the goal of the work, yet commonly appear *as a result of work*. By simplifying under performance pressure we do not act randomly. Rather, we put work in, and get novelty out: a new, useful prediction or connection emerges that was not originally expected. Thus, it is not the case that either one knows one’s goal and there is no novelty in getting there, or one does not know it and the only way to get there is by accident. Rather, there is a third way to novelty.

Several important comments follow. First, we need not explain why simplification under performance pressure leads to novel, useful things in science that were not directly sought. For now, we may simply take it as a grand fact.

Second, importantly, this simplification does not make science as a whole simpler but rather more complex. As new theories connect between previously unconnected facts, new predictions and new questions arise. The more knowns there are, the more they interact and expand our ability to ask yet new questions. Thus, I argue that simplification under performance pressure leads to both novelty and complexity.

Third, simplification and performance function together. As statisticians or investigators in machine learning know, it is useless to make a model that predicts a given set of data points perfectly if the model is overly complicated, as it is useless to set up a model that is very simple but has nothing to do with the data. A balance must be maintained between fit to data and model elegance, and to maintain it is an art.

Indeed, the desires for simplicity and for performance are *conflicting:* at the time when Galileo originally favored the Copernican over the Ptolemaic system, he did it *despite* the fact that the former fit the data a *little* worse, and because of the fact that it was *much* more parsimonious. Indeed, later scientific research showed that the more parsimonious model was far more *improvable*.

The development of mathematics gives us a similar picture. It happened once and again in history that pure mathematicians working on the principles of aesthetics or parsimony have produced things that years later were found to have unexpected utilitarian value (Wigner 1960; Hamming 1980; Byers 2010). Indeed, the power of operations other than the test of performance in the growth of mathematical and scientific knowledge has been amply demonstrated. We see it in simplification or parsimony, elegance or aesthetics, symmetry, pattern completion and analogy (Wigner 1960; Hamming 1980; Byers 2010). *I use the word “simplification” in this paper in a very broad sense to refer to all such variants and the creative force they represent*. Note also that in both mathematics and science, we operate with a network of concepts. We connect between ideas to create a fuzzy, new idea, distill a fuzzy new idea to its essence, and pursue the consequences of a distilled idea to new connections (Christos Papadimitriou and Umesh Vazirani, personal communications). Thus, novelty arises from the network, not from random, point-like changes. This network change is driven by both simplification and performance, and we can see that it leads to complexity, novelty and improvement.

The evolution of technology is also illustrative. What is simple appears in many different technologies. The concept of a disc appears in the potter’s wheel, in wheels for transportation, in a round table, and in the cross section of a tree trunk. The concept of a sharp edge appears in a stone tool, a peg, and even a shingle roof. Once we generate a functional but elegant object in one context, it is going to have the inherent capacity of working well in future, different contexts.

I argue here that, also in biological evolution, simplification and performance pressure, and not accidental mutation and performance pressure, drive complexity, novelty and advancement. This new theory has an advantage over the previous one. When we rely on simplification under performance pressure, we rely on something that we can see to be central to other creative processes.

A key aspect of simplification is that it allows us to circumvent the problem posed earlier: how can mutation do anything useful, how can it be anything besides accidental, without “awareness” of the environment and the macroscale phenotype? The solution is that work that simplifies local connections in the genetic network requires no Lamarckian knowledge of the macroscale phenotype and the environment, and can take place in the germ cells. That is, while local simplification and gene duplication operations take place in the germ cells, natural selection evaluates the organism as a complex whole, and together these two forces lead to novelty. This allows us to replace the concept of accidental mutation with a concept of *non-accidental mutation that is useful yet not Lamarckian*, and thus to replace the traditional notion of random mutation as the ultimate source of new heritable information in evolution.^14^

### Where Simplification and Performance Pressure Happen

In addition to simplification pressure at the genetic level and performance pressure at the organismal level, each of the two may have, at its own level, the other on the other side of the coin. For example, in an ecological community, each species is pressing to produce more of itself and at the same time is undoing the growth of others, thus pressing to simplify the ecological network. The same could be said of a gene that comes to replace another in the course of evolution by usurping the other’s role, a phenomenon called “genetic piracy” by Roth (1988) (see also Wagner 2014). The ecological example above clarifies that simplification can be as natural as differential survival. In fact, here simplification and differential survival are two sides of the same coin: inasmuch as the making more of one entity means making less of another, the performance of any one entity puts simplification pressure on the network, and this principle may apply both to the ecological network and to the genetic network. It is also noteworthy in this regard that a gene that is extensively used (performs well) and is therefore highly expressed may, due to mutational mechanisms, be more likely to be duplicated. This will be relevant in Sect. 5.5.

### Simplification and Novelty: Summary

We have seen in this section ideas according to which simplification under performance pressure leads to novelty, complexity and high performance. We have seen that these ideas are fundamentally connected to network-level evolution. The next section will examine various relevant empirical facts.

## The Problem of Innateness

The evolution of innateness has been one of the oldest and most mysterious problems in evolution (Lamarck 1809; Darwin 1859; Duerden 1920; Waddington 1942; Lorenz 1966). We have seen the evolution of innateness at the genetic level in the cases of gene fusion and the evolution of alternative splicing patterns, and we will now see it at the organismal level. Although the empirical facts to be discussed are known, they have not yet been used to make a new argument about the nature of the evolutionary process. I will focus on examples from the evolution of behavior, thus complementing past literature on novelty, which has focused on morphological evolution (Müller 1990; Müller and Wagner 1991; Müller and Newman 2005; Moczek 2008; Wagner and Lynch 2010; Hall and Kerney 2012; Hallgrímsson et al. 2012; Peterson and Müller 2013).

I will first cover innateness from multiple angles in Sects. 4.1–4.11, and then discuss the emergence of novelty in detail (Sect. 4.13). We will see that improvement, innateness, novelty and stabilization all come together as different aspects of one process. At the end of Sect. 4, both the consistency with past literature on novelty and the contributions to it will be clear (Sect. 4.16). Readers interested in the molecular level may note that it will be revisited in Sect. 5.

### The Problem of the Preexistence of High-Level Mechanisms

The ability of pointer dogs to point at the prey in a statuesque manner is to a large degree innate (Arkwright 1902). How did this instinct evolve? To argue that a sequence of random mutations of small effects has built up the behavior from scratch such that it has always been instinctive and never learned is unappealing: Would breeders have recognized slight inborn tendencies to point at the beginning of the evolutionary process involved and, without regard for the outcome of any training, base their artificial selection on these differences? And if training were important in the evolution of pointing, one may expect that the highly evolved abilities of the animal to learn would have masked out presumed mutations of slight effect for an *independently developed* instinct. All would be much more understandable if we consider that a trait that previously required learning through reward and/or punishment has become emancipated in the course of evolution from these external cues.

Consider the evolution of migration. In an instinctive and automatic manner, a young common cuckoo (*Cuculus canorus*) takes off in the fall from its breeding grounds in Scandinavia, flies thousands of miles to its wintering site in Central Africa and then returns in the spring (Willemoes et al. 2014). How did this complex suite of instincts get started in evolution? Both Darwin (Romains 1883) and Wallace (1874) hypothesized that the breeding and wintering grounds gradually became separated and the distance between them increased; that originally, the animals were tracking seasonal changes in resources over short distances as a direct response to the environment; and that in time this behavior became habitual and instinctive (Romains 1883; Wallace 1874; see also Woodbury 1941). To assume that the migratory instinct evolved afresh, independently of the behavior that came before it, brings up the same problem as in the case of the pointer dogs: the pre-existence of an evolved, general-level mechanism (in these cases, the brain) that is able to respond adaptively to environmental changes and was presumably involved in the original phenotype.

In an experiment designed to capture the evolution of innateness (Waddington 1953) (see also Waddington 1956, 2006, 1959; Bateman 1959a, b), Waddington took *Drosophila melanogaster* flies and exposed their pupae to a heat shock. As a result, a fair number of the flies that developed showed a particular vein pattern on their wings called “crossveinless”.^15^ He then bred the crossveinless flies to form the next generation of the experiment and repeated this procedure of heat shock and selective breeding over the generations. As a result, the percentage of crossveinless flies increased over the generations and, beginning at generation 14, a small percentage of flies started showing the new vein pattern without exposure to heat shock, that is, innately^16^ (Waddington 1953). The intriguing experimental outcome was that this trait became innate, *even though no selection for such innateness was performed*.

To explain this outcome, called “genetic assimilation” (Waddington 1953), Stern (1958) (see also Falconer 1960) proposed a model based on traditional principles. The model assumes the preexistence of alleles that make independent contributions toward a certain sum, such that if the sum surpasses a certain threshold, the trait of interest is exhibited. Furthermore it makes certain assumptions about the initial frequencies of alleles and the normal and experimental conditions thresholds that make it so that, prior to selection, the trait of interest (e.g., crossveinless) is exhibited in practice only under experimental conditions (e.g., heat shock), whereas post selection it is exhibited under both experimental and normal conditions, and thus the trait can be said to have become innate. However, despite the mathematical crispness of this model, taken literally, it means that every trait that is to become innate has its own set of additive alleles that preexist and provide the potential for that trait to become innate as is. That is, there are additive alleles that, if they surpass a threshold, build a brain that points, and there are different sets of additive alleles lying dormant in birds for every possible migration route, such that each set builds a brain for a particular route if it surpasses a certain threshold.^17^ Indeed, Waddington himself rejected this model (Waddington 1958, 1961), because it did not apply to the complex natural cases that motivated the problem. Here, I will provide another explanation for innateness based on interaction-based evolution.

### Modularity and Innateness are Caused by Simplification

As Waddington (1942) alluded to, when an emerging module is released from the influence of an element inside the organism, the result is seen as modularization; and when it is released from the influence of an environmental factor, the result is seen as the evolution of innateness. In Sects. 2.2 and 3.1 I argued that simplification is connected to modularity and innateness: the formation of modules streamlines the developmental process and involves emancipation of an emerging module from complex influences, both internal and external. Indeed, *simplification leads to modularity and innateness.*

Approaching the topic of innateness equipped with the theory of gradual network change presented here, it is useful to distinguish between two important phenomena that I will call “emancipation” and “acceleration.” Emancipation refers to the fact that units (modules or elements) can be copied and the connections between units can gradually evolve such that a unit can be subjected to different regulation than that of its source copy. Acceleration refers to the idea that the coming together of units under one control simplifies development locally while absorbing novel phenotypic meaning from the changing context. Both these aspects of network level evolution, discussed in Sect. 2.2, will be clarified here with the help of examples, and both figure into the explanation of innateness to be given in the following sections.

### The Evolution of Innateness is More Common than We Realize

In an idealized view of the crossveinless experiment, we can think of the crossveinless trait as qualitative (present or absent) and assume that it is the same in the beginning of the experiment as it is at the end. The only thing that evolves under this assumption is the propensity to produce it. In this case, we may simply use the word “emancipation” to describe what happens to the crossveinless trait when it comes to appear without the environmental trigger. But crossveinless is an extreme, chosen for its simplicity. In nature, when the evolution of innateness or emancipation takes place, the trait that is to become innate also evolves at the same time. For example, in cases of ritualization, a non-signaling behavior is gradually released from its context and becomes used as a signal (e.g., an egg-fanning movement becomes a showing-the-nest signal, Tinbergen 1951; see Sect. 4.6) (Huxley 1914; Whitman 1919; Armstrong 1950; Tinbergen 1952). As Tinbergen noted, those ritualized traits that are emancipated are usually traits that have already changed much from their original form; and we would not have been able to make a connection between the signal and its origin if it were not for the fact that, at least in some cases, there happened to be a transitional series betraying the connection between the two, such as in the threat posture of the Manchurian crane (*Grus japonensis*) (Lorenz 1935; Tinbergen 1952). In other words, there exists a continuum of differences between the non-innate ancestral and the innate derived traits, that ranges from no difference, to a great difference that obscures the connection between the ancestral and the derived; and cases at the former end of the spectrum are rare.

I argue that this is precisely the problem with observing innateness. Darwin and other early naturalists believed that what is habitually performed due to environmental triggers over the generations gradually impresses itself on the hereditary constitution of the species and becomes innate and emancipated from the environment, and that this is explained by the laws of use and disuse, or Lamarckism (Romains 1883; Darwin 1859). I argue that such automatization does happen in general but is often hard to see because of the difference between the ancestral and derived traits, *and that it is network-level evolution and not Lamarckism that is responsible for it.*

### Evolution of the Whole as a Whole in Innateness

The fact that a trait changes as it becomes innate allows us to examine the evolution of a complex whole, while involving not only emancipation but also fusion and acceleration.

While I have been using the term “innate” without qualification so far, it is useful to note that there is no strict separation between the innate and the non-innate. Learning itself is enabled by instinct (Lorenz 1965; Gould and Marler 1986). No trait develops in a manner that is independent of the innate nature of the organism, and no trait develops entirely independently of the environment, when the latter is broadly construed (Lehrman 1953). Therefore, rather than speaking of “innate” and “non-innate,” we realize that there is a continuum between traits developed more directly and quickly (“innate”) and traits that require more unfolding that involves more interactions with the environment.

When we consider this continuum as it applies to a given organism, we should consider that it evolves as a whole—the process of development evolves as a whole. Then, we can bring the ideas of gradual network evolution to bear on it (Sect. 2). I argue here that the evolution of innateness arises as a result of the “chunking” or modularization of a previously complex part of the network—what were previously independent elements each under a different control have now become simplified or combined into a singe unit. Although it may seem that this simplification accelerates development in the course of evolution toward the final trait, in general, development is not accelerating toward the final trait as it was before, but rather toward what that trait has in the meantime changed into, and therefore nothing is being accelerated strictly speaking. Therefore, it is the signature of the previously less innate that we generally see in the current more innate, rather than a direct facsimile. There is no Lamarckian transmission that takes a developed or a learned trait and makes it innate. As argued in Sect. 2.2, to use a metaphor, in an Archimedes screw, water is moved along the shaft even though each point in the screw only rotates at its own level. So in evolution, the non-innate does not itself become innate—the phenotypic does not become genotypic—but rather evolutionary action at each level of biological organization takes place within that level, while accelerating development.

Indeed, we know that the adult form influences the evolution of the earlier stages of development: there is selection on the adult form, and therefore there is selection on earlier stages of development to lead to a well-performing adult. When considered from a traditional standpoint, this trivial point turns into a problem because *development is a complex process*, and traditional evolutionary theory is not equipped to deal with a complex process. Traditional evolutionary theory cannot conceive of a complex evolutionary change that modifies the developmental process as a complex whole: it does not have a sense of acceleration^18^ or an emphasis on emancipation, and therefore when a trait appears earlier in development that seems to relate to one that used to come later in development (e.g., innate migration relates to earlier, learned migration), it absurdly has to invoke an evolution of that trait afresh, absent any connection to that which it obviously relates to. While Gould attempted to address this problem, he did so by breaking the whole again into parts and arguing that the timing of appearance of one part or a developmental process in and of itself can be moved earlier or later in development (Gould 1977), which is a very limited explanation that does not address the range of phenomena discussed here.

I have presented, in contrast, a view of the evolution of the whole as a whole. Instead of the evolution “afresh” idea that arises from a traditional perspective, this view raises the notion of acceleration as described: *The quicker arriving at an evolving developmental outcome has the appearance of the evolution of innateness, thus involving interaction-based, network-level evolution in innateness. We have seen this in the case of the TRIM5-CypA fusion and the evolution of alternative splicing patterns at the molecular level, and will see it now in many examples at the organismal level.*

### Example: Pointing in Pointer Dogs

Pointing in pointer dogs will serve as an example of the importance of the evolution of the whole as a whole in innateness. I propose that selection has operated on the outcome of the training, favoring hunting dogs whose behavior following training was more pleasing to their owners, specifically in stopping upon discovery of the prey instead of chasing it further. However, since innate tendencies guide the learning, this selection has operated indirectly on innate tendencies, favoring dogs with the right set of innate tendencies that were more naturally inclined to learn the right behavior. In particular, I hypothesize that there exists among animals a widespread tendency to heed sudden changes; that in pointer dogs, this tendency has been strengthened in the course of evolution, in particular by heightening nervousness; and that other instincts have become at the same time adjusted to direct it productively, helping the dogs to learn to pause and freeze at the sight of prey. Over the generations, the learning task has become more and more natural to the dogs, and the amount of learning required has decreased, until today, the dogs require only minimal training, and they sometimes point at objects innately without any learning, as Darwin observed (see F. Darwin ed. 1909). Thus, selection for an improved outcome of the learning was accompanied by an acceleration of the learning and, ultimately, innateness. This hypothesis already has the advantage that it explains not only the evolution of the pointer dog instinct complex but also the nervousness syndrome that often afflicts these dogs: heightened nervousness helps them heed sudden changes, and when not properly compensated for results in the nervousness syndrome. Note that, while Grandin and Deesing (1998) argued before that pointing relates to nervousness, their discussion seems to assume that pointing and nervousness are traits with separate genetic causes that happen to be connected through genetic linkage, which suggests a spurious connection. In contrast, the hypothesis proposed here connects pointing and nervousness at a deep level, with the help of the holistic view of interaction-based evolution. There are no genes dedicated to pointing per se: pointing is a system-level phenomenon that emerges from a suite of interacting instincts and learning.

In an exceptionally inspiring chapter, Papaj has already argued that what is at first learned, over evolutionary time can come to be learned more quickly until it eventually becomes innate (Papaj 1993). In this respect, his hypothesis is similar to the above. However, lacking the ideas of interaction-based evolution, and treating instinct and learning as separate elements, he tried to create a model of a traditional kind, and admitted that the model failed to provide an explanation, because the evolution of innateness came out of an artificiality built into it (Papaj 1993). In contrast, the hypothesis presented here allows us to preserve Papaj’s intuition but in a natural way: it holds that selection has affected interacting instincts that guide the complex process of development and learning through a process of network-based evolution, and network evolution in turn involves acceleration and emancipation—an increase in the innate abilities. In addition, interaction-based evolution also explains why innateness and stereotypy are deeply intertwined (see Sects. 4.10, 4.11), which Papaj admits his model does not (Papaj 1993).

To reiterate, I propose that the process of evolution toward a better outcome of the learning leads at the same time to a quickening of the learning and that ultimately, a new innate trait appears, because what enables the organism to reach a better outcome through learning is that it is naturally inclined in the right direction. The organism “gets it” more, naturally and inherently, because underlying interacting instincts are being shaped. Thus, *improvement comes together with innateness.*

Importantly, the evolutionary process that shapes the network of underlying instincts can be seen as uncovering better “principles” that guide the learning (and more generally, development)—emerging underlying elements that organize a preexisting complex more simply. Consistent with Sect. 3 and with later sections (see Sect. 4.13), viewing things in terms of such principles leads us to a new prediction regarding novelty: the evolution of the new innate and the new and improved adult form will come together with the production of new, beneficial things *that were not selected for in and of themselves but arose as corollaries or windfalls of figuring out the right principles*. Improvement, innateness, *and generalization*—or the emergence of useful novelty not selected for—come together. As an example, backing in pointer dogs^19^ may have evolved as a “corollary” of pointing—an unintended but desirable outcome.

### Elements of Network Evolution: Chunking, Duplication, Emancipation, Cooption and Rigidification

As noted, ritualization is an evolutionary process that occurs when a behavioral element is gradually emancipated from its original use as it becomes coopted for use as a signal in the course of evolution (see, among other sources, Huxley 1914; Whitman 1919; Armstrong 1950; Tinbergen 1952 and further references below). For example, when a bird is about to hop or take flight, it bends its legs, lowers its breast, raises its hind parts and sometimes its tail, folds its neck and brings its head back almost to the shoulders, while slightly expanding its wings, so that the whole body is like a tight spring ready to be released for jumping, at which instant the legs straighten, the breast and hind parts line up with the direction of the jump, and the neck is stretched forward (Daanje 1951). Daanje argued that, from this movement, various signals have evolved. For example, when the male turkey displays to the female, it raises the hindparts a bit, raises and spreads its tail, folds its neck and brings its head back almost to the raised back feathers, and partly spreads its wings downwards. This posture imitates that of the jump in several elements, except that the legs are not bent, the tail and wing movements are exaggerated, and the posture is kept frozen for a while (Daanje 1951). Thus, a behavioral pattern which is widespread taxonomically and which originally had a mechanical function has evolved into a signal that is expressed in new contexts independently of the context of expression of the ancestral trait.

Another example of ritualization is the way that the male three-spined stickleback (*Gasterosteus aculeatus*) shows the nest entrance to the female. According to Tinbergen, this movement was derived from the egg fanning movement (Tinbergen 1951), which again demonstrates a shift from one context to another.

As we have just seen, ritualization requires *emancipation* from one context and *cooption* to another. Critically, these are operations of network evolution. The gradual release of an element from one context concomitant with the subjecting of it to another context involves two aspects of the organism at once and is inherently an interactive operation, well-described by modules moving in a network.

Baerends’s work on nest building, egg laying and offspring provisioning in the sand wasp *Ammophila adriaansei* (*campestris*) (Baerends 1941) demonstrates clearly that behavior is underlain by a network of modules. A normal behavioral sequence of the wasps is as follows: Build a nest; close the entrance temporarily with soil; fly away and hunt a caterpillar; carry the paralyzed caterpillar back; reopen the nest; put the caterpillar in; lay an egg; close the entrance again, this time with greater care. Now build another nest and repeat the entire process so far. Now return to the first nest; open the closure; make an inspection visit. If the egg has hatched and the nest is in order, close the entrance, and now bring 1-3 caterpillars in succession. Repeat this second phase for the second nest. Now return to the first nest, open the closure and make an inspection visit. If all is in order, bring 3-7 caterpillars in succession; then make an especially careful final closure of the nest entrance. Repeat this third phase for the second nest. Now build another nest, and repeat all from the beginning. Furthermore, if, in the first inspection described above, the egg has not hatched, the wasp may build another nest at that time. It can manage 4 nests at a time, with offspring at different ages at each nest requiring different amounts of provisioning (based on information obtained in inspection visits). If a nest has been disturbed, the wasp may abandon it.

A computer programmer would instantly recognize that the sand wasp’s behavior is *an algorithm with subroutines* (see flow chart in Fig. 1). The most parsimonious description of this behavior involves activation of the same behavioral modules or subroutines, such as “carry a caterpillar to nest” or “perform an inspection visit” in different contexts, and the different contexts, namely the different stages of laying and provisioning, themselves consist of different combinations of lower-level behavioral modules (Lorenz 1981).

Interestingly, Tinbergen wrote that the process underlying emancipation was not known, though it must somehow involve natural selection (Tinbergen 1952). The present theory highlights how correct he was to emphasize that unknown. At once we can understand the inability of traditional evolutionary theory to explain and connect with classical ethology: A network is defined by interactions. The evolution of a network is the evolution of a complex whole. The conceptualization of evolution based on traditional theory encouraged a one-trait-at-a-time type of thinking and was not suitable for discussing network evolution and the transfer of an element from one context to another (Gould and Vrba 1982; Gould 2002). Importantly, Tinbergen also noted that there is no point during emancipation at which a behavioral element stops belonging to its original function and starts belonging to a new function (Tinbergen 1952). Rather, as in the verbal model of network evolution discussed in Sect. 2.2, the change of context and meaning is gradual.

Let us now think about the evolution of a network such as described by Baerends. Obviously, elements were not added to it in the form in which they exist today. For example, the construction of a well-shaped nest with a cell at the end had been preceded by a less involved modification of the environment. Also, elements were not appended in the course of evolution at the end of the behavioral sequence. That is, if “build nest”, “make closure,” “hunt caterpillar,” etc., are denoted *a*, *b*, *c*, etc., then it is patently obvious that the stages of evolution did not proceed in the following sequence: *a*, *ab*, *abc*, etc., or else absurdities arise such as not laying eggs until a certain point in evolution, performing an inspection visit before the existence of a foraging stage where information from this visit is used, etc. This means that the behavioral sequence was reorganized in the course of evolution and/or new elements were added at internal positions in the sequence. It follows that elements that came after positions into which new or preexisting elements were inserted, or from which preexisting elements were removed or translocated, must have been emancipated from their previous triggers (namely the completion of the behavioral steps that used to come before them) and subjected to new triggers (the completion of the behavioral steps that come before them now). Finally, we would not assume that each repeating element or subroutine evolved afresh in its entirety for each instance in the sequence in which it is used. This means that there has been a copying of routine calls, or, to use more generic terms, *copying and differentiation of modules*. Thus, operators of network evolution—emancipation, cooption, copying and differentiation of modules—have been involved in the evolution of sand wasp behavior.

Another example showing the insertion of elements at internal points in a sequence and sequence reordering is Lorenz’s study of display sequences in surface feeding ducks (Lorenz 1958). Lorenz found about 20 behavioral elements, of which different combinations make different display sequences in different species and even within the same species. Lorenz (1958) describes the study of three different species, the mallard, the European teal (*Anas crecca*) and the gadwall (*Anas strepera*), which share the following 10 elements:

- 1. Initial bill-shake
- 2. Head-flick
- 3. Tail-shake
- 4. Grunt-whistle
- 5. Head-up-tail-up
- 6. Turn toward the female
- 7. Nod-swimming
- 8. Turning the back of the head
- 9. Bridling
- 10. Down-up movement

He then presents some display sequences (where one element follows another in quick succession) for each of the three species. For the mallard:

- 3,2,3
- 1,4,3
- 5,6,7,8

For the European teal:

- 3,2,3
- 10
- 4,3,2,5,6,8

For the gadwall:

- 4,3,2,3
- 5,6;10,6

(the semicolon mark between sequences 5,6 and 10,6 in the gadwall means that they are fused at high excitation, which suggests, by connection with many other observations, that we are observing them in the midst of a process of evolutionary fusion; Lorenz 1958).

The sequences above are obligatory and innate. Hybrids produce their own sequences. This clearly shows network-level evolution in the sense of reorganization of modules, emancipation and fusion at the level of sequences of fixed action patterns (FAPs).

Critically, this example and the previous ones show us that *the picture that we obtain by looking closely at evolution at the phenotypic level mirrors what the molecular biological and genomic revolutions have taught us about the genetic level:* at both the molecular and organismal levels, network-level evolution is key (Wagner and Lynch 2010; Lynch et al. 2011, 2015; Müller and Wagner 1991; Hallgrímsson et al. 2005; Müller 2007a; Moczek 2008; Schlosser 2015). And network-level evolution is much better understood with the help of the principles of interaction-based evolution, including cooption, emancipation, acceleration and simplification.

Fusion, like emancipation, is also a network-evolution operation. While Baerends’s and Lorenz’s examples above demonstrate it at the level of sequences of FAPs, fusion can also generate elements at a lower level, namely the level of the single FAP; though—again mirroring the genetic level (see discussion in Sect. 2.4)—there is no sharp boundary between the single FAP and sequences thereof. One telling example was the inciting ceremony in ducks (Lorenz 1941/1971, 1958, 1966) described in Sect. 2.1: The movements of threatening the neighbors with the neck and of returning to the mate, originally triggered by separate environmental stimuli, have gradually fused over the course of evolution into a stationary neck-over-the-shoulder display triggered as one, while becoming emancipated from the presence of neighbors. These movements originally constituted territorial behavior, with an indirect, implied meaning of pair-bonding and team work; and *as they fused, the pair-bonding meaning crystallized and moved to the fore* (Lorenz 1941/1971, 1958, 1966).

Many related examples exist. The tendency to attack a neighbor when in one’s own territory and flee from the neighbor when in the neighbor’s territory is indeed a very general one, spanning birds, fish and mammals. In some fish, the neighbors exchange chase and be-chased turns, coincident with crossing the territorial boundary (Lorenz 1981). In the fire-mouth cyclid, *Cichlasoma meeki*, this chase and be-chased movement has become a highly rhythmic oscillation—it has become stereotyped. The fusion of the previously separately triggered back-and-forth movements in this species is revealed when one fish suddenly loses interest and disengages yet the other continues oscillating (Lorenz 1981). Evolution, in this sense, is a process of automatization.

### Generalizing Beyond Ritualization: The Automatic Nature of Instinct

The following examples not only show that fusion and other elements of network evolution extend beyond ritualization but also demonstrate the automatic nature of instinct. Consider, for example, the pecking instinct in domestic chicks. This fixed action pattern (FAP) is present at birth and consists of three main elements: lunging the head, opening and closing the beak, and swallowing (Lehrman 1953). Since we would not assume that these three elements of the fixed action pattern have each evolved from scratch in the context of this FAP, we are forced to assume that they have been fused.

The classic example of a FAP—egg rolling in the greylag goose (*Anser anser*)—also shows fusion. Upon seeing an egg placed by the side of its nest, the goose stretches its neck in a particular fashion, places its beak over the egg, and then slowly rolls the egg back into the nest while performing balancing sideways motions with the beak to prevent the egg from slipping from the side (Lorenz and Tinbergen 1938/1970). This seems like an insightful sequence of operations, but in fact, when the egg is quickly pulled out from under the beak while in motion, the goose will continue to roll the remaining nothingness all the way to completion and tuck it under (Lorenz and Tinbergen 1938/1970), again showing the automatic nature of instinct.

Indeed, it is implicit in Barlow’s definition of the FAP that the FAP is a fusion of elements in general (Barlow 1977). Barlow defined the FAP (which he renamed “modal action pattern,” MAP) as a behavioral module *usually indivisible* but *made of elements that appear individually elsewhere.* It is quite informative that Lorenz (1958) had suggested that “perhaps all behavioral patterns” arise from fusion such as seen in the inciting example.

### Ritualization Shows All Elements of Network Evolution in One

Interestingly, a single case of ritualization often exemplifies multiple or even all of the following characteristics: emancipation (also: routinization, autonomization, or evolution of innateness), cooption, fusion (chunking, or welding), increased efficiency, exaggeration (or “caricaturization”), schematization, simplification, stereotypy, automatization and rigidification (Huxley 1914; Whitman 1919; Armstrong 1950; Tinbergen 1952). Traditional theory has only offered to explain one or another of these phenomena in separate from the others. For example, Maynard-Smith and Harper (2004) suggested that stereotypy evolves because it standardizes competition. This suggestion not only ignores the fact that stereotypy exists also in non-signaling instincts where such competition is absent, but also ignores the common co-occurrence of the many elements above-mentioned. In contrast, the principles discussed in this paper unify these observations under one umbrella, as outlined below:

- *Emancipation*: Emancipation (the release of a module from previous influences), or the evolution of innateness, is clearly demonstrated by the examples above, and has been addressed here as a part of interaction-based (or network-based) evolution. The same is true for chunking—the fusion of modules—which is also a part of network-based evolution.
- *Simplification*: A fundamental concept in ethology is that of the “sign stimulus”—the stimulus that elicits a FAP. “Sign” means “simple”: the sign stimulus obtained its name from the fact that the animal attends only to a very limited part of the situation that we know it to be capable of perceiving through its senses. It attends to a parsimonious summary of the situation—a key. Yet the simplicity of the key is only relative: it is still a complex whole, a pattern involving relations between elements (Tinbergen and Kuenen 1939; Lorenz 1939; Krätzig 1940; Tinbergen 1951). For example, the gaping response of nestling *Turdus merula* as soon as they open their eyes can be directed at a model consisting of a mere three discs that touch each other. However, it is preferentially directed toward one of the discs that bears the right size-relation to another disc, such that the two together can be interpreted as a simplified schema of head and body (Tinbergen and Kuenen 1939). As another example, an abstract cross-like model (including symmetrical anterior and posterior “wing” edges, and central short and long protrusions perpendicular to them) elicited an escape response from young birds, but only when it was moved in the direction of the short end of the cross, as only in this case the short end can be interpreted as a short neck, which is the case for birds of prey (Lorenz 1939; Krätzig 1940; Tinbergen 1951). In other words, *an abstract combination of elements* is the evolved key. Now, Tinbergen argued that evolved rituals, which are themselves stimuli eliciting behavior in others, have been “schematized” through evolution and are evolved sign stimuli (Tinbergen 1952). Thus, in retrospect, his statement is consistent with the claim made here that rituals (and I will add the reception of signals, the “innate releasing mechanism,” or IRM; Lorenz 1981) have been evolutionarily simplified under performance pressure to their complex essence.
- *Exaggeration (or “caricaturization”)*: Ritualized signals are often exaggerated, as in the case of throwing the neck over one shoulder and then the other during inciting in the golden-eye (Sect. 2.1). Although it has been suggested that exaggeration has evolved under natural selection for visual clarity, one may ask whether organisms really need such a degree of clarity.^20^ I argue instead that exaggeration is related to “caricaturization” or “schematization” (terms used in the literature) and the final touch of perfection (Sect. 3.1), and evolves by active simplification under performance pressure (Sect. 3), not by minute economical considerations.
- *Stereotypy*: Stereotypy—or the lack of variation between individuals in a certain trait, or even between different instances of the behavior in the same individual—is another prominent aspect of rituals. According to interaction-based evolution, stereotypy is an inherent aspect of evolution, as will be discussed in Sect. 4.10.
- *Cooption*: Cooption is inherent to Tinbergen’s definition of ritualization, as noted (a non-signaling behavior is coopted as a signal) and is also a crucial part of interaction-based evolution.

Thus, interaction-based evolution provides a more parsimonious view of ritualization than traditional theory. This provides both additional support for interaction-based evolution and an improved conceptual understanding of ritualization.

### Recapitulation: Network Evolution and Phenotypes

In the model of network evolution (Sect. 2.2), I argued that two genetic elements that previously were regulated by two separate lines of controls and had to be separately expressed before coming together in an interaction can, over evolutionary time, gradually come together under one control and even fuse to form a new gene. In this process, there is not only emancipation (since one or both of these elements is emancipated from what previously controlled it) but also a sense of automatization, innateness and acceleration, as the emerging unit is no longer constructed from its elements by developmental interactions but rather has been evolutionarily accelerated into a ready-made unit or gene. We can now see that, like the genetic-level examples, the organismal-level examples that demonstrate emancipation and cooption also demonstrate the evolution of innateness, automatization, and acceleration, as expected. In the pecking instinct of domestic chicks, for instance, the lunging of the head, the opening and closing of the beak, and the swallowing, have been fused together. Therefore, the last two elements follow the first one automatically now, even though they must have been originally triggered separately by the environment. Furthermore, this welded instinct appears soon after hatching, and perhaps even in the embryo to some degree (Lehrman 1953; Kuo 1932a, b), demonstrating the evolution of innateness and acceleration. Similar points can be made, for example, about egg rolling in the greylag goose or the inciting ceremony in ducks (Sect. 2.1).

### Stereotypy and the Evolution of Complex Phenotypes: The Process by Which Phenotypes Become Fixed

Interaction-based evolution argues that selection evaluates individuals as complex wholes (Livnat 2013). Unlike selection on additive variance (Fisher 1930), it argues that genetic interactions are at the core of the adaptive evolutionary process. Unlike the shifting balance theory (Wright 1931, 1932), it argues that genetic interactions are not formed by random genetic drift but rather transient genetic combinations are continually subject to selection. This kind of process has not yet been modeled mathematically. However, Livnat (2013) has made the following verbal argument regarding it. If selection acts on transient, complex combinations of interacting alleles across loci, then the information relevant for adaptive evolution under selection must be non-local: adaptive phenotypic change does not appear first in one individual and then spread to all, but rather forms at the population level through the concomitant spread under selection of many interacting alleles across loci (Livnat 2013). As some alleles spread in the population to fixation, the phenotypic variation that used to be caused by the sexual shuffling of these alleles must also gradually disappear, and thus, with regards to this variation, individuals must *gradually* become more similar to each other over the generations (Livnat 2013). This implies that stabilization accompanies the evolution of adaptation in general, rather than requires a separate force for stabilization of a trait per se, implemented by accidental mutation and natural selection^21^ (Stearns 2002). I called this effect “convergence” at the population level (Livnat 2013) (note that this usage of the term is distinct from the usual meaning of convergence in evolution).

If this is correct, then we should see a continuum of phenotypic fixedness corresponding to the formation of traits, with different traits lying at present at different points along that continuum. Some traits, still early in the process of formation, will appear as less stable or stereotyped, and others, at later stages in the process, will appear as more so. Thus, under interaction-based evolution, *stereotypy can be seen as an indication of the degree of evolutionary progress of a trait.*

An example is provided again by the pointers. Darwin wrote that the hunting behaviors that characterize the pointers are basically innate, and that the only difference between them and “true instinct” is that they are “less strictly inherited” in that there is variation in the individuals’ “degree of inborn perfection” and therefore in the extent to which they require training (Romains 1883, p. 237). Indeed, pointing in a statuesque manner, backing other dogs and other hunting-relevant behaviors have all been observed to occur often in pups that have not had the opportunity for learning by instruction, imitation or experience, and they are not exhibited immediately or to the same degree in all pups (Arkwright 1902). When training is required, the amount of training required is small: the trainer only guides the dog toward expressing what it already has a strong natural tendency to express (Arkwright 1902). The existence of variation in pointing behavior becomes natural from the perspective of interaction-based evolution: if traits evolve from fuzzy to sharp, if they are gradually stabilized, as discussed by Livnat (2013), then this simply means that pointing is still in the process of formation and has not yet become perfectly innate.

In light of the above, interaction-based evolution provides a distinctly different view than the traditional one on the evolution of stereotypy. Those who previously tried to explain stereotypy argued that signals must be clear, that stereotypy makes them clearer, and that they are selected for this extra clarity in a traditional process of selection (Mayr 1974; Barlow 1977).^22^ However, I argue, first, that the clarity-based approach lacked parsimony from the beginning, because stereotypy is a property of instinct in general, not just of signaling behavior specifically; and second, that the clarity-based approach is unnecessary: the degree of uniformity is an outcome of the temporal nature of the process. This degree is in general associated with the point that the trait has reached along the spectrum of formation, and we observe that it varies across traits because we are witnessing traits at different stages of formation. Even if uniformity per se can be of value, the traditional focus on uniformity as a separate end obscures the general point: stabilization is not in and of itself a separate target of selection as traditionally perceived; it is an inherent part of interaction-based evolution.^23^

Hinde’s comparative study of displays in finches demonstrates several of the points above (Hinde 1955). First, the same trait may vary more or be more stereotyped in one species than another. Second, demonstrating the evolution of FAPs from interactions, most displays in the finches studied by Hinde are not yet fixed action patterns but rather are poses whose elements tend to occur together statistically. Third, of the different displays, the one which is particularly rigid, stereotyped and emancipated (the female soliciting posture), is also the one that is far more widely shared, and therefore more ancient, than the other, still variable displays, in accord with the prediction above that stereotypy is indicative of the evolutionary stage of formation of a trait. Another, in-depth example showing the evolution of different degrees of fixedness, and that stereotypy is a concomitant of the evolution of adaptation—the evolution of decoy-nest construction in sand wasps—will be given in Sect. 4.12.

### Interaction-Based Evolution Provides a Single Explanation to the Different Aspects of Innateness

“Innate behavior” or “instinct” has been used to mean different things in the literature (Papaj 1993):

1. Independence: It has been used to refer to behavior that is *independent of interactions with the external environment* like learning or experience. Such independence is especially clear when a behavior is present from birth, as for example in the pecking of domestic chicks (Lehrman 1953).^24^
2. Stereotypy: Innate behavior has been referred to as *stereotyped* (also, “fixed,” “constant,” “rigid” —“robotic;” Papaj 1993).
3. Sharedness: Innate behaviors have been found to be homologizable between species and therefore useful for taxonomy. In other words, they are *shared between species*, with some species-specific characteristics (Lorenz 1981; Heinroth 1911; Whitman 1898, 1919).

The fact that these three aspects are connected empirically is clear.^25^ But why are they connected? Tinbergen’s tone regarding the empirical connection between points 2 and 3 above was that of surprise (Tinbergen 1951, p.191). Barlow discussed stereotypy in the context of the clarity of signals (Barlow 1977), which neither addresses stereotypy in non-signaling behavior nor explains the connection between stereotypy and independence. And Papaj’s model was unable to connect stereotypy and independence (Papaj 1993).

However, interaction-based evolution connects the different aspects of innateness. First, traits evolve by a process of convergence as defined (see Sect. 4.10; Livnat, 2013)—a process of stabilization which begins at a state of high variance and eventually leads to the evolution of uniformity and therefore stereotypy (Livnat 2013). Since this process takes time, it makes it so that stereotyped elements are older and more widely shared than elements not yet stereotyped, while implying that stereotypy evolves across related species in parallel. *This point connects the sharing of a trait between species with stereotypy* (aspects 3 and 2 above, respectively).

Second, I have argued that interaction-based evolution works in the long-term through simplification under performance pressure. This can not only make one intra-organismal module independent of another, but also make it independent of environmental factors (see Sect. 4.2). Therefore, the process of interaction-based evolution leads not only to stereotypy but also to increased independence from environmental factors (1). *This ties together independence, phylogenetic sharedness and stereotypy.*

Interaction-based evolution also explains two other important aspects of innateness. One is that the fixed action pattern (FAP) often and perhaps always consists of fused elements (Lorenz 1958) (see Sects. 4.6, 4.7), which aligns with the fact that fusion is an outcome of network-level evolution. The other is the issue of circuitous vs. accelerated development, which will be discussed later (see Sect. 4.14), and which will clarify the connection between evolution and learning.

### Evolution from Fuzzy to Sharp: Examples of the Evolution of Complex Phenotypes

In this paper, we have seen that examples of the evolution of complex phenotypes fit with the view of interaction-based evolution: they demonstrate cooption and emancipation, stabilization and stereotypy, and evolution from fuzzy to sharp. But perhaps most intriguingly, they *speak to the arising of novelty in a way that is consistent with network-level evolution*.

I will first discuss in detail the example of decoy construction in sand wasps, taken from Evans’s classic ethological work (Evans 1966a, b). Readers who wish to skip this example may continue directly to the final and most important organismal level example to be discussed here—that of egg retrieval by backward walking in the nightjar (Sect. 4.13)—where I will demonstrate all the elements discussed in this paper in one, with an emphasis on the central point of novelty.

To make a nest, the Bembicinae (previously called Nyssoninae) sand wasps dig a tunnel of many body-lengths, at the end of which they build a cell or a complex of cells, where they place their offspring and the prey on which the offspring will feed (Evans 1966b). They have natural enemies—two taxonomic groups of flies (the bee flies, Bombyliidae, and miltogrammine flies, Sarcophagidae) and two taxonomic groups of parasitic wasps (the cuckoo wasps, Chrysididae, and “velvet ants,” Mutillidae)— that parasitize their nests by laying there, and whose larvae either takes up valuable resources or destroy the larvae of the sand wasp. Parasites of the former two groups seek the nests of their hosts by sight, and of the latter two groups by touch and odor—they tap the soil with their antennae while searching for their target (Evans 1966b). Across the sand wasps we find various techniques of hiding and concealment as well as a decoy construction technique (Evans 1966b).

Our example concerns the decoy construction: Some species of sand wasp dig a false burrow (or multiple such burrows) next to the real one and leave it (or them) open, while leaving the real nest burrow closed. Various parasites have been observed to either lay their eggs or linger in the decoys. Evans hypothesized that false burrows originated in a behavior that had a different purpose—that its origin is in cooption—and that the process of the evolution of digging false burrows was a process of improvement through stereotypy, together with emancipation (see also Tsuneki 1963).

He based his hypothesis on the following facts. Very commonly across the Bembicinae, the wasps close the entrance to their nest from the outside before leaving it, either temporarily with a small amount of soil before leaving temporarily for provisioning, or with a large amount of soil at the final closure before leaving for good; and both within and outside of the Bembicinae, species have been observed where individuals obtain soil for nest closure mostly from several or one particular spot/s around the entrance. In this case they leave behind a small pit or pits of a size that depends on how much a particular spot was used. The tendency to take soil from a particular spot or spots appears to relate to environmental conditions, where individuals quarry soil for closure when loose soil is not as easily available (Evans 1966a), though it also has a genetic component (Evans 1966a). The more soil is quarried from a particular spot, the bigger the pit left behind. In *Bembecinus neglectus*, for example, most individuals took the soil for closure from several particular spots around the entrance, so that a ring of small depressions was left behind. But some took it mostly from one particular spot, which formed a “depression or short “false burrow” up to 1 cm deep” (Evans 1966b, p. 137).

Now, in the genus *Bembix*, which typifies the advanced behaviors of the Bembicinae, we see species with decided false burrows. Consider three species: *Bembix amoena*, *B. texana* and *B. sayi*. In *B. amoena*, false burrows are of irregular occurrence and spatial pattern and are relatively short, and the burrowing is still almost always associated with quarrying soil for closure. Most individuals obtain soil for the initial closure by scraping a small amount of soil from each side of the nest entrance, and occasionally from a particular spot or spots, creating short false burrows that stretch in a direction of 90 degrees or a bit less left or right of the direction of the true nest, obliquely into the ground. These short false side burrows varied in length from barely perceptible to 2 cm long, with two exceptional cases observed at 3 and 5 cm long (Evans 1966b). Evans (1966b) noted that, in one population, about half of the nests had no such false side burrows, about a quarter had one, and the rest had two such false side burrows at one time or another. In addition, individuals also collected soil from opposite of the nest entrance, most often, but not always, for the final closure (which requires more soil), which resulted either in a furrow going through the mound of soil opposite the true nest (a mound resulting from the excavation of the true nest), or in a burrow running under that mound obliquely through the ground. The former appeared a considerable number of times, (Evans 1966b, p. 280) and were of varying length, between 1 and 7 cm long. The latter appeared rarely (Evans 1966b), with only three cases noted, at 1.5, 2 and 3 cm long. Evans (1966b, p. 281) noted that two of these were made *following* final closure in a manner similar to that of *Bembix sayi* (see below), which seems to suggest that these two rare instances occurred not in the service of obtaining soil for closure.

Besides the burrows themselves, the manner and timing of construction of the burrows was also variable of course (Evans 1966a, b). The burrows on the sides, when they occurred, occurred sometimes along with the initial closure and sometimes along with later closures. The back burrows and furrows, when they appeared, appeared often along with the final closure but sometimes along with earlier closures, and the final closure did not always involve them. While burrows were sometimes revisited and expanded, they were sometimes accidentally filled while making a closure. Likewise, the spatial pattern resulting at the end was variable, with 0 or 1 or 2 side burrows and 0 or 1 back burrow or furrow (Evans 1966a, b). Note that, although the soil was generally taken for closures, parasites were distracted by the false burrows that resulted (Evans 1966a).

In *B. texana*, construction of false burrows is more or less regular, and the soil is not used for closure. Typically, individuals construct one short but relatively persistent false burrow on each side of the entrance, right after the initial closure is made (Evans 1966b). The method of construction is not yet entirely stereotyped, with some individuals digging one burrow and then the other, and others alternating in digging both (Evans 1966b, p. 325).

In *B. sayi*, construction is invariable and emancipated: all females dig one strong back burrow 4–22 cm long under the mound after completion of final closure (which also means that the soil is not used for closure) (Evans 1966b, a).

The three species above exemplify certain general trends (Evans 1966b, a). The primitive cases of false burrows, where the burrow is but a small pit, are unreliable and irregular in appearance. The “transitional” case of *B. amoena* shows burrows appearing in a notable number of cases but still rather irregularly. They are often longer than “small pits” but are still relatively short and vary greatly in length. In the advanced cases, the burrows appear with greater regularity and are substantial. In *B. sayi*, which makes the longest burrows, the burrows appear invariably in all individuals and at a regular time. There is an association between stereotypy and completeness of the burrow (Evans 1966a, b).

In addition, there is an association between stereotypy and emancipation of burrow-making from its previous cause (Evans 1966b, a). That is, limited quarrying in the service of obtaining soil for closure is a widespread phenomenon, and tends to be irregular in occurrence; whereas, in contrast, regular burrows are the result of burrowing for its own sake, an operation not used for closure, and are constructed at regular times before or after closure, depending on the particular species concerned. In some of the advanced species where burrow-making is thus emancipated, the wasps refresh or fix burrows that have been destroyed (Tsuneki 1963), which further clarifies that they are programmed to maintain a certain pattern of false burrows. The above characteristics are also associated with increased regularity of the spatial pattern of the burrows, with each of the different emancipated species having its own idiosyncratic characteristics of construction (Evans 1966b, a).

Furthermore, not only have emancipated burrows never been observed in species that lack closures, but in addition, a careful examination of the data in Evans (1966b) shows that, even though they are no longer used for closure, emancipated back burrows are temporally associated with final closures, and emancipated side burrows are temporally associated with initial closures, which supports the fact that the origin of emancipated burrows is in closure-making.

Thus, evidence clearly supports the predictions of interaction-based evolution. The evolution of false burrows *originated in cooption—in emerging high-level interactions between preevolved elements like digging, quarrying and making closures, and environmental elements like sand conditions*. That starting state was one of high variance in behavior and outcome within and between individuals. Evolution then proceeded *from fuzzy to sharp:* through a process of convergence and gradual stabilization of the trait as a whole *toward a stable, emancipated and clock-work-like state*. The process was one of improvement together with and at the same time as stereotypy and emancipation. Note that it is not the case that complete but irregular burrows evolved first, and then were stabilized. That is, stereotypy, or uniformity, is not an outcome of a force of stabilizing selection separate from the selection for the adaptation itself. Rather, *stabilization and improvement evolve together as two aspects of the same coin—as inherent concomitants of the adaptive evolution of the whole as a whole*, as predicted by interaction-based evolution.

### The Emergence of Novelty in the Evolution of Egg Retrieval by Backward Walking

I will now discuss the final and most important organismal-level example that puts all of the elements of the theory discussed here together, while emphasizing the central point of the emergence of novelty. This is the example of the evolution of egg retrieval by backward walking in the nightjar (*Caprimulgus europaeus*) and other species, which applies to eggs that have rolled far outside the nest. Before we can understand it, we must first see what the shifting motion in birds is.

The shifting motion in birds is ancient and involves rolling an egg with the beak until it reaches under the body. The egg may have thus gotten in between other eggs and stirred them, and the egg sides that are pointing up are thus changed (Tinbergen 1960). Shifting may be needed to ensure even temperature distribution to the eggs (Caldwell and Cornwell 1975), and is performed upon arrival at the nest, or when the tactile stimulus provided by the eggs while brooding is not satisfying, or spontaneously after a long spell of quiet brooding (Tinbergen 1960). In terns, the shifting motion will move an egg about 2–3 inches.

Coming back to our case of egg retrieval, in terns (e.g., *Onychoprion fuscatus*), the general situation is as follows (Watson and Lashley 1915; Tinbergen 1960): If they notice an egg lying several inches outside of the nest, they leave the nest right away to it. However, they have an aversion toward being far from the nest, induced by their brooding state, and as they move away from the nest, they slow down, sometimes turning around and returning to the nest without having reached the external egg. But sometimes they do get to the egg, stopping short of it just close enough that they can reach it with the beak and apply the shifting motion to it, which rolls the egg until it is under the breast.

As they shift the egg, they sit down on it to incubate it, but only for a short time (indeed they may at this point be dissatisfied with the tactile stimulus and/or with being outside of the nest). The moment they notice the nest again they stand up and walk to it. In the process, the egg has moved about 2–3 inches toward the nest due to the shifting motion.

Having returned to the nest and started brooding the eggs there, they soon notice the external egg again, venture out toward it again, and repeat the process, and the egg moves 2–3 inches again toward the nest. Thus, after several trips, the egg finds its way back to the nest.

The behavior that results in the egg being moved back to the nest is clearly unstructured. The brooding of the external egg outside of the nest and the back-and-forth trips show lack of insight or “analysis of the situation as a whole” (Watson and Lashley 1915, p. 83), as the different actions taken in the situation are under the proximate control of different preevolved instincts. In accordance with Tinbergen (1960), these instincts are competing with each other for expression: the desire not to leave the nest, the desire to return to the nest, the desire to brood eggs, and the desire and ability to shift an egg. Also, as Marshall noted for the common tern (*Sterna hirundo*) (Marshall 1943), there is much variance in the behavior and its outcome, with eggs sometimes being rolled back into the nest and sometimes not, and this variance is thought to reflect both individual variance and situational factors (Marshall 1943).

In other birds, however, such as greylag geese (*Anser anser*), black-headed gulls (*Chroicocephalus ridibundus*) and nightjars, the bird walks straight up to the egg, puts the beak over it as it would in shifting, but instead of incubating the egg there, it then walks backwards all the way to the nest in one shot while shifting and dragging the egg under its beak (Kirkman 1937; Tinbergen 1960).

According to Tinbergen, this egg rolling observed in nightjars and other birds evolved from shifting and other elements of the situation (Tinbergen 1960). Indeed, the fact that the birds are using a shifting motion while walking backward (even though rolling with the wing would have been much more efficient) together with the fact that shifting is ancient, supports this hypothesis (Tinbergen 1960). In fact, Tinbergen notes that the very controversy about whether egg retrieval is an independent adaptation or a by-product of a confluence of instincts in different species shows its route of evolution (Tinbergen 1960).

The argument that this backward walking behavior appearing in nightjars and other birds evolved from a situation akin to that of the terns exemplifies several elements of interaction-based evolution in one: The trait has evolved from fuzzy to sharp; from unstructured and inefficient to structured and efficient; from variable and unstable to stable, stereotyped and “rigorous.” In addition, we also see emancipation in it: the return back to the nest originally required the visual stimulus of seeing the nest, but now is triggered automatically as soon as the shifting motion begins and requires no turning-around to the nest. We also see fusion: the going-to-the-egg and the coming-back-to-the-nest legs have been fused together in one sequence unleashed by the stimulus of seeing the external egg for the first time, whereas previously they were two separate legs each triggered by its own visual stimulus. The whole situation has been *simplified*, the path has been straightened up. In fact, the simplification has been the creation of a method from a previously non-methodical occurrence, when all the while the whole evolved as a whole, not by the addition of independent elements one at a time.

On top of all of the above, one topic deserves a special emphasis: novelty. The example of egg rolling shows clearly that different instincts or elements have the inherent ability to come together into new, useful interactions that together can achieve what had been unachievable before by any one of those instincts alone. Twice we see that this coming together of pre-evolved elements into useful, high-level interactions breaks a barrier in terms of being able to do something that could not have been done before. First, the confluence of instincts for shifting, brooding and returning to the nest effectively allows the egg to be returned to the nest after several trips, even if in a haphazard way, when none of these instincts by itself is capable of achieving this, nor did any of them originate due to pressure for such egg retrieval. The second barrier broken was this: the invention of backward walking while shifting allows retrieval of the egg in time that is proportional simply to the distance to the egg, whereas the haphazard way only allows retrieval in time that is quadratic in distance. This improvement allows nightjars to retrieve eggs from many yards away, which would not have been effectively possible in the case of terns (indeed terns retrieve eggs from only several inches away). Thus, emancipation and fusion have created a behavior that now applies to a broader range of situations than the ancestral traits applied to. Indeed, some of these birds now dwell in beaches where eggs can indeed be blown away by wind a great distance.

These breakings of barriers exemplify novelty. The source of the novelty is the inherent ability of elements to come together into new and useful high-level interactions. These elements come together first in a haphazard state. Their complex interaction then serves as a substrate for simplification under performance pressure, where new such elements will be formed. I propose that this cycle is the heart of the evolutionary process; and that it is simplification under performance pressure that is responsible for this inherent usefulness of elements—for their propensity to come together into new, useful interactions that they have not been directly selected for.

While consistent with previous works on the evolution of novelty, as will be discussed in Sect. 4.16, this point provides an understanding of novelty in evolution that is completely different from and not reducible to the traditional notion of accidental mutation and natural selection. The novelty that drives evolution here arises from the coming together of high-level “modules,” i.e., from the network as a whole. It is not a local, “misspelling”-like change at the genetic level. This demonstrates the key point that the source of invention in evolution does not need to be accidental mutation. Instead, I argue that *non*-accidental mutation and natural selection together process information gradually, and the source of novelty in evolution is the resulting inherent ability of elements to come together in useful high-level interactions, an ability due to simplification under performance pressure.

Watson and Lashley (1915) saw that the outcome of the haphazard mode of egg retrieval was not intentional. They noted that the egg rolls in the direction of the nest simply because the bird is oriented directly away from the nest as it reaches the egg, so that the shifting motion happens to bring the egg a bit closer to the nest each time. From this they concluded that the egg finds its way to the nest by lucky happenstance. But this lucky happenstance is a far cry from the traditional notion of novelty in random mutation. First, it is not a local accidental mutation that invents, but rather the process starts with high-level interactions, and gradual network evolution creates a new trait from this source. Second, a deep new question arises. Should we call this source of novelty “randomness” or “lucky happenstance” at the phenotypic level, and say no more? There is logic to the present situation that goes beyond the purely coincidental. That is, although the egg rolls to the nest only because of the bird’s orientation, it is oriented in this way because it is walking straight up to the egg from the nest. Each action—walking straight up, shifting and returning—is efficient and elegant, and together they create a situation that, while haphazard, is not purely random, but can be seen as a fuzzy sort of “*shifting in an extended nest*.” Thus, a confluence of instincts, each useful in and of itself, together give rise to something useful that is different from each of them, but which at first can only appear in a roundabout, highly variable, even though not purely accidental, fashion. As such, it serves as material for evolutionary simplification and streamlining, which ends up creating something that can be useful in contexts that go beyond the one that originated it.

It is intriguing that an unqualified notion of the accidental does not sufficiently explain novelty here. When we apply this way of thinking to other cases of cooption, we will see that, individually, they may appear more or less accidental than the above case; but they are not, in general, “pure coincidences.” This, together with the question of how exactly simplification under performance pressure leads to inherently useful elements, contributes a new dimension to the science of novelty (Gould and Vrba 1982; Müller 1990; Müller and Wagner 1991; Gould 2002; Moczek 2008; Wagner 2014).

While others have discussed the possibility of cooption being a source of novelty (Gould and Vrba 1982; Hallgrímsson et al. 2012; Müller 1990; Müller and Wagner 1991; Schlichting and Pigliucci 1998; West-Eberhard 2003; Müller and Newman 2005; Moczek 2008; Peterson and Müller 2013), this possibility has not been sufficiently distinguished from the view of accidental mutation—cooption has often been treated as an *accidental* event and *another* source of novelty *in addition to* accidental mutation (Williams 1966; Gould and Vrba 1982; Gould 2002). In contrast, interaction-based evolution argues that cooption is neither an accidental event nor another source of novelty in addition to accidental mutation. Rather, *the joint action of non-accidental mutation and natural selection gradually paves the way to both genetic and phenotypic cooption* through network-level evolution. Thus, neither mutation nor cooption are random in the traditional sense even though they produce surprising things, and they are not separate sources of novelty but come together as two inseparable aspects of one process. In other words, cooption is at the heart of the process of interaction-based evolution and is built into this process.

Thus, while in the traditional view, new genetic information arises by accident at a specific point in space and time, according to interaction-based evolution, novelty is an outcome that arises over time at the network level from the coevolutionary change of many elements. *While the drivers of these local changes are not random, these changes still interact with each other globally in a surprising way.* Surprise, or novelty, exists, but it is not a mere direct effect of dice rolling.

It is noteworthy that the tern situation is based on conflict, or competition between tendencies. The bird, on the one hand, acts as though it wants to reach up to the egg and incubate it, but on the other hand as though it wants to remain in the nest. It is also noteworthy that there is individual variation in the overall behavior, and indeed, there may be different ways of increasing the probability of success. One way may be to approach the egg without hesitation. Another may be to get back to the nest without delay once the egg has been shifted. There is an inherent conflict in the situation. Both tendencies have something to contribute, but they are conflicting. To strengthen one at the expense of the other may be harmful. One may suppose that evolution needs to take a modest though complex step: to find a solution for returning to the nest immediately and only after reaching the egg while engaging it with the beak. This can be achieved, for example, by overcoming the tendency to incubate but only while standing outside of the nest. The relevant rule to evolve, “incubate in the presence of eggs AND when standing in the nest” is simple but non-linear. In performing this evolutionary step, the convergence process described by Livnat (2013) may lead to the crystallization of the commonality between successful individuals while resolving the conflict inherent in the situation, making evolution a process of conflict resolution.

The example also shows us, of course, that the whole is greater than the sum of its parts and that the organism evolves as a whole. Conceptualizing evolution in this way provides an answer to the many inconsistencies that arise from the accidental mutation framework and the related conceptualization of evolution as a one-step-at-a-time type of process, such as the fact that the latter often lead us to surmise difficult evolutionary transitions leading to complex adaptations where the intervening steps are not adaptive in and of themselves (Wright 1931, 1932; see also Moczek 2008; Hallgrímsson et al. 2012; Arnold 2003; Müller and Newman 2005; Peterson and Müller 2013; Kaji et al. 2016).

Finally, a connection similar to the one pointed out between punctualism and gradualism in morphological evolution (Müller and Streicher 1989; Müller and Wagner 1991; Moczek 2008; Hallgrímsson et al. 2012; Peterson and Müller 2013) can be seen in the above behavioral example. Suppose that, as the underlying instincts evolve and are being emancipated and adjusted, the balance of tendencies gradually changes such that the tendency to incubate the external egg while outside of the nest falls below the tendency to return to the nest, while the tendency to return remains balanced with shifting, so that returning and shifting are expressed together. In that case, these tendencies may evolve gradually, while the new trait may arise punctually: it may be possible for a bird species to evolve backward-walking retrieval as a whole and rather rapidly, causing an evolutionary “phase transition” at the level of the observed behavior. Backward walking will then appear first in one individual, then in another, then more and more—it will arrive “like the rain.” Thus, punctualism is better understood when we start thinking of it in terms of network evolution, as an outcome of gradual network-change trends that interact with each other (Müller and Wagner 1991; Moczek 2008; Hallgrímsson et al. 2012; Peterson and Müller 2013).

### How Evolution Learns: Circuitous Versus Accelerated Development

The example of the evolution of egg retrieval highlights a fifth aspect of innateness. The terns are not *learning* to retrieve the egg, yet their haphazard emergent behavior which results in egg retrieval does not seem to fit the term “innate.” There is another aspect to innateness, and that is the degree to which a behavior develops straightforwardly and quickly in an endogenously driven fashion. For example, though an emerged butterfly takes to the air after it finishes preparations (like drying up its wings), this behavior of taking off is not actually driven but only triggered by finishing preparations, and is otherwise endogenously driven. In contrast, the behavior which results in egg retrieval in the terns arises circuitously and at a high level, from a meeting of different inborn behavioral elements as well as environmental factors which play a more inherent role in inducing the behavior: the visual stimuli and conflicting instincts do cause the retrieval of the egg. This high level meeting of modules, both internal and external, now serves as the source of evolution from fuzzy to sharp, at the end of which a new innate module, consisting of a combination of the previously independent elements, will arise (that of backward walking). This aspect, namely, how quickly, straightforwardly and endogenously a behavior (or a trait in general, including morphological traits) arises in development is important for evolutionary acceleration: In the initial circuity there is a potential for “straightening up,” there is a potential for simplification that will lead to the further breaking of barriers (e.g., substantially faster egg retrieval). The environmental factors play a more inherent role in inducing the behavioral outcome in the tern situation than in the butterfly situation, which means that, in becoming emancipated from them in the course of evolution, the life form is “learning” from the environment evolutionarily. That is, it now produces more endogenously what the environment helped to produce before. *I argue that this emancipation is the intergenerational “learning” that is done by evolution*, drawing an analogy between evolution and learning (see more in Sect. 5.7).

It is interesting that those who have tried to define innateness often seemed to mean that, in contrast with learned behavior, innate behavior is in a sense “predetermined” (Papaj 1993). In other words, in innate traits, the fit with the environment is predetermined, as opposed to learned behavior or morphological plasticity, where the fit is “acquired.” However, notice that this predetermined fit *is* adaptation. In a system-first view of evolution, the evolution of innateness, or automatization, *is* the evolution of adaptation. And, according to interaction-based evolution, the evolution of adaptation involves network-level evolution and the acquisition of a new phenotypic meaning as a result of the changing context in which modules are embedded.

### The Engine of Evolution

Interaction-based evolution argues that the process whereby a population converges on an adaptation (Livnat 2013; Sects. 4.10, 4.12, 4.13) is a process that converts information from a less orderly to a more orderly state. It proceeds from a fuzzy to a sharp, well-working and stereotyped state. However, evolution is not *only* a fuzzy-to-sharp process, in that the fuzzy source must first arise. The progress from fuzzy to sharp is therefore only a half of a cycle of the “engine of creativity” that is evolution. The other half is that previously made sharp elements come together at a high level to make the new fuzzy source (e.g., the different instincts in the tern situation come together into a disorderly form of egg-retrieval), from which new sharp elements can be made (e.g., backward walking).^26^ I argued that simplification under performance pressure connects the two parts of the cycle. The simple elements it creates not only are improvements but also come together in new complex interactions which serve as the raw material for the next round of simplification. Thus, novelty arises not from accident, but from evolutionary work.

### How Interaction-Based Evolution Contributes to Past Literature on the Evolution of Novelty

We can now see how interaction-based evolution confirms previous, pioneering literature on the evolution of novelty. Even though the latter focused on the evolution of morphology (Müller and Wagner 1991; Moczek 2008; Wagner and Lynch 2010; Peterson and Müller 2013), whereas the observations above pertain to the evolution of behavior, both conclude that network-level evolution is key to the evolution of novelty, and furthermore agree on multiple fundamental points that follow from this: First, cooption is central to novelty, as are elements of network evolution like duplication, fusion etc. (Gould and Vrba 1982; Müller 1990; Müller and Wagner 1991; Schlichting and Pigliucci 1998; West-Eberhard 2003; Hallgrímsson et al. 2005; Müller 2007a; Hallgrímsson et al. 2012; Wagner 2014; Lynch et al. 2015). The emergence of a “byproduct,” where a trait evolved for one purpose comes to serve another, is not a side-issue but absolutely central to evolution (Sects. 2.2, 3.4, 4.5, 4.12, 4.13, 4.15; Müller 1990; Müller and Wagner 1991; Müller and Newman 2005; Moczek 2008; Peterson and Müller 2013). Second, cooption is needed for the otherwise inexplicable incipient stage of a novel, complex adaptation^27^ (Moczek 2008; Hallgrímsson et al. 2012; Arnold 2003; Müller and Newman 2005; Peterson and Müller 2013; Kaji et al. 2016). Third, the common distinction in the literature on novelty between two stages of evolution—the modification of an existing character and the formation of a new character (Gould and Vrba 1982; Müller and Wagner 1991; Müller and Newman 2005; Wagner and Lynch 2010; Hallgrímsson et al. 2012)—corresponds to the two stages of the evolutionary cycle discussed here (see Sect. 4.15). Fourth, network-level evolution—the evolution of a complex whole—allows for the conversion of gradual to punctual change via the passing of thresholds (we have seen this in the egg retrieval discussion, Sect. 4.13; Waddington 1957; Müller and Streicher 1989; Müller and Wagner 1991; Moczek 2008; Hallgrímsson et al. 2012; Peterson and Müller 2013).

At the same time, interaction-based evolution also contributes to this literature. First, as mentioned, while the latter focused on morphology, in the above I have provided complementary examples from the evolution of behavior. Second, even though past literature has recognized the centrality of cooption for novelty and identified phenomena that facilitate cooption, such as gene duplication, “weak regulatory linkage” and more (Ohno 1970; Kirschner and Gerhart 2006; Gerhart and Kirschner 2007; Lynch 2007), it has not considered the possibility that there is a fundamental reason underlying the inherent ability of biological entities to be coopted, or that this reason connects to non-accidental mutation. This paper has proposed that simplification under performance pressure actively generates cooptability (see Sect. 3.4). Furthermore, it has proposed that simplification under performance pressure connects to non-accidental mutation as will be summarized in Sect. 5.2. Third, while this paper agrees with past literature on the distinction between the two stages abovementioned, it does not assume that the modification of existing characters can be accounted for by the traditional view of adaptation based on accidental mutation and natural selection, and that only the arising of a novel character cannot. Instead, it argues that the mutations that underlie network-level evolution throughout both stages are non-accidental and enable simplification under performance pressure; that this simplification under performance pressure is what makes a character cooptable while modifying it; and that the two stages thus come together in one unified process: one stage actively leads to the other.

Thus, the present paper bears on a major gap in traditional evolutionary theory. Multiple authors (Gould and Vrba 1982; Hallgrímsson et al. 2012; Müller 1990; Müller and Wagner 1991; Schlichting and Pigliucci 1998; West-Eberhard 2003; Müller and Newman 2005; Moczek 2008; Peterson and Müller 2013; Lynch et al. 2011) have recognized the importance of cooption at both the molecular and organismal levels. Graur and Li called it “the paradigm of molecular evolution” (Graur and Li 2000, p. 304). However, traditional population genetics focused on trying to explain the accumulation of slightly beneficial changes toward improvement in the same adaptation, not on how an element evolved for one purpose can later become useful for another (Gould and Vrba 1982; Müller and Newman 2005; Wagner and Lynch 2010). This paper proposed that there is an active reason underlying cooptability—simplification under performance pressure—and that this active process offers an alternative explanation to many biological phenomena at both the genetic and phenotypic levels besides economical considerations under accidental mutation and natural selection.

## A New View of the Evolutionary Process

In this section I will revisit the molecular level from the perspective of interaction-based evolution in light of the concepts proposed so far. I will clarify the nature of mutation and raise directions for future research regarding it.

### Interaction-Based Evolution at the Microscale

Livnat (2013) introduced interaction-based evolution at the microscale. I will now reiterate some key points of the latter and then show how it is connected to the present paper.

Livnat (2013) argued that the mutations that drive adaptive evolution under selection result from continually evolving, complex biological processes and are therefore affected by genetic interactions across loci. Conceptualizing this in terms of information flow, it means that there is a flow of information from the alleles affecting the mutation into the mutation itself (Livnat 2013). The schematic figure that describes this nature of mutation (Fig. 2a) is much like that which would represent gene interaction and regulation, except that the outcome of the action in this case is genetic change. Importantly, “mutation” here is broadly construed to encompass not only DNA mutations but also epigenetic changes.

Moving to the population level, we see that the outcome of a mutational event in one generation—namely the mutation itself—can serve as an input into mutational events at later generations. Therefore, mutations create a network of information flow across the genome and the generations (Fig. 3) (Livnat 2013). This immediately opens up a new way of thinking on how the genome can evolve as a cohesive whole (Mayr 1963; Livnat 2013).

This view connects the problem of the role of sexual reproduction in evolution to the nature of mutation. The layman’s intuition has been that, since natural selection acts on individual variation, the vast number of different genetic combinations generated by sex facilitates adaptive evolution. However, this answer has been incomplete from a theoretical perspective because, just as sex puts together these combinations, it also breaks them down. In other words, what is the point of presenting so many different combinations to the test of selection, if they are not heritable? Here, the computational nature of mutation (Livnat 2013; Livnat and Papadimitriou 2016a, b) changes the situation.

Since mutation combines information from multiple loci and writes the result of the combination into one locus—the locus of the mutation—it allows information from genetic combinations to be transmitted to future generations even though the combinations themselves are transient (Fig. 2b) (Livnat 2013; Livnat and Papadimitriou 2016b). Intriguingly, such information flow through mutation enables a situation where sex generates a vast number of different individuals, selection evaluates each individual *as a complex whole*, and information from that individual as a complex whole is passed on by mutations precisely in accord with the individual’s fitness (Livnat 2013).^28^

In this way, interaction-based evolution connected the problem of the role of sex in evolution, the empirical nature of mutation, and more, while raising predictions and directions for future research. Among these predictions are the following: (i) there is a genetic relatedness in mutational tendencies, and these tendencies are of fundamental significance for the process of adaptive evolution; (ii) genes have a dual role: they encode both information that is relevant for their performance and information that is relevant for genetic change; and (iii) there exists a vast genetic activity in germ cells that is essential for adaptive evolution. Thus, while it is clear today that there are complex influences on mutation, Livnat (2013) argued that they are of fundamental importance for evolution. The present paper builds on the previous one in an important way: it relies on non-accidental mutation to enact simplification under performance pressure, as demonstrated in Sect. 2.

### Connections Between the Microscale and the Macroscale

Simplification under performance pressure and network-level evolution connect to non-accidental mutation as follows: (i) They *make it possible* for mutation to be non-accidental without reversing the one-way function from genotype to phenotype (Sect. 3.4). (ii) They *remove the conceptual need* for local genetic accident to be the source of new genetic information: because novelty arises at the system level from emerging interactions between units, the fact that the drivers of local genetic changes are non-accidental is not contradictory to the emergence of novelty, because these changes still interact with each other globally in surprising and beneficial ways (Sects. 2.2, 3.4, 4.13). (iii) They *require* mechanisms of mutation to drive gradual network-level evolution in a syntactic way, including mechanisms of fusion, deletion, duplication and more (Sects. 2, 2.3, 2.2, 5.4, 5.5).

However, as in past evolutionary theory, there is still a gap to be filled between genetic and phenotypic change. Long-term phenotypic fusion such as that of the neck and body movements in the inciting ceremony in ducks may require multiple events of gene rearrangement and point mutations. Therefore, understanding the causes of a gene fusion such as that of *TRIM5* and *CypA* is of course a far cry from providing a detailed genetic account for a long-term, phenotypic fusion. In addition, interaction-based evolution does not yet provide a detailed list of mutational mechanisms. However, it does provide a small taste of such a list, including discussions of transcriptional promiscuity in germ cells (Sect. 2; Livnat 2013), genetic interactions as affecting mutations (Sect. 2; Livnat 2013), mechanisms of fusion and duplication as have been and will be discussed in this paper (Sects. 5.4, 5.5), and more.

A clarification is now required on the connection between the microscale and macroscale. In both the microscale and the macroscale views of interaction-based evolution developed by Livnat (2013) and here respectively, there is a sense of chunking: On the microscale, information from multiple loci comes together in an event of genetic (or epigenetic) change. On the macroscale, genes as well as phenotypes can become fused in the long-term. However, the flow of information from the alleles at multiple loci affecting a mutation into the locus of the mutation does not mean that the mutation replicates and transmits the combination of alleles that influenced it as is. Rather, the situation is partly analogous to that of a neuron, which sends a signal to further layers in the neural network generated from the transient combination of inputs that it received, but does not replicate that combination as is. That is, Fig. 3 depicts the information flow that is the moment-to-moment workings of evolution; and it is many such events of information flow, at many loci, in many individuals, that come together into a network of information flow that *gradually* leads through the generations to the conditions permitting genetic- and organismal-level chunking and cooption in the long-term (e.g., gene fusion, the evolution of alternative splicing patterns, or phenotypic fusion; Sects. 2, 2.1, 2.3).

### The Two Ecologies Working Together: The Ecology of Energy and the Ecology of Information

I will now put the arguments of Livnat (2013) and of this paper into one philosophical picture. A machine has several aspects: First, it is a finite, unchanging structure that repeats its operation over and over again, performing the same “trick.” Second, we tend to think of a machine as something that operates harmoniously and whose parts have been conceived to fit each other harmoniously. Third, novelty or “out of the box” thinking is the antithesis of machine-like behavior.

Now, the traditional idea of natural selection and random mutation is machine-like in the first sense: it is one trick that repeats itself indefinitely without changing its own fundamental nature. That is, random mutation occurs either as an error during replication or for other accidental reasons, and natural selection either accepts it or rejects it. The repetition of this operation is traditionally supposed to be responsible for all of life and every innovation in it—a belief that I have argued against.

Here, I will draw the distinction that the writing of mutation postulated by interaction-based evolution (Livnat 2013) is not machine-like in any of the above senses. First, the writing itself evolves and its evolution is fundamental to its operation—its operation is not repetitive (Livnat 2013). Second, the workings of evolution are not devoid of internal conflict but rather are based on it, as will be discussed shortly. Third, the production of novelty is at the essence of evolution. (Notice, however, that while evolution is not machine-like, its products are machine-like: evolution is a process of automatization, as the observations show; Sect. 4).

What is the nature of mutation then? Let us consider the nature of regular traits first. Take locomotion for example: we share with bears the fact that we have four limbs; but unlike bears we are habitual bipeds; and each of us may have a specific leg length and muscular details slightly different from those of others. In other words, a trait consists of widely shared and generally defined characteristics along with more specific and more narrowly shared characteristics, up to and including individual differences. According to interaction-based evolution, the mutation-writing phenotype has the same meta-structure as the performing phenotype (Livnat 2013). It consists of generally defined and widely shared characteristics (for example, the long-term trend of the movement of genes out of the X chromosome in *Drosophila*; Vibranovski et al. 2009), along with more specific and narrowly shared characteristics up to and including individual differences in mutational tendencies (Livnat 2013). This meta-structure implies that mutational tendencies are more similar the more closely related the entities under consideration are (Livnat 2013). Furthermore, it implies that the nature of mutational mechanisms can be conceptualized by analogy to ecological interactions: The writing of mutations happens not according to a fixed “rule” but by the ever evolving “rules of the jungle.” This “jungle” is a complex one consisting of DNA and other biomolecules. The actors in it—the genetic influences on mutation—meet in an individual due to sexual reproduction, and genetic changes happen in accordance with the usual tendencies of the actors, their individual characteristics and the particular combination they appear in in the given situation. All the while, the actors themselves slowly evolve in the long-term. Thus, when we talk about the workings of mutation, we are not talking about a harmonious, repetitive operation of a single mechanism. Instead, we are talking about the workings of an “ecology,” except that the outcome is remembered not in terms of energy transfers such as food-web interactions but in terms of symbolic changes in genomes: it is an ecology of information.

According to this picture, the biological world has two facets to it, two “forces”: one that is due to biological interactions that make their mark through differential survival and reproduction; and one that is due to biological interactions that make their mark by influencing genetic change—by the writing of mutations (Livnat 2013). These latter biological interactions are not limited to molecular mechanisms operating inside the germ cells, but involve also anything else that affects the writing of mutations, such as mechanisms of mate choice and of the sexual shuffling of the genes (Livnat 2013).

These two forces come together in the individual: the selection of individuals determines which alleles will be passed on, and the writing of alleles determines which alleles will be there in the first place. Thus, selection and writing are equally influential forces, and they both participate in changing the genetic and phenotypic nature of the organism and thus of themselves. While each of these forces has some long-term (phylogenetically shared) tendencies, each is oblivious to the present, immediate workings of the other: the intra-organismal writing of mutations that takes place at the present moment is shielded from the external workings of natural selection that takes place at the present moment and vice-versa, even though the consequences of each will eventually affect the nature of the other. In that sense, evolution arises from a conflict, or a process of negotiation, between these two fundamental forces; and what happens in the long-term must be more or less congruent with both.

### A Balance of Continually Evolving Mutational Forces is Responsible for Genetic Change

I argued above that the writing of mutations is analogous to an ecology. An ecology is a system of conflicting forces, where each species presses to produce more of itself while at the same time undoing the growth of others. And indeed, when we examine molecular evolutionary changes, we often see that long-term processes result from a balance of forces in the short-term.

Consider tandem gene duplication. A gene that is duplicated at tandem experiences an increased chance not only of being further duplicated but also of losing a copy, due to the nature of the mutational mechanisms of tandem duplication and deletion (Graur and Li 2000). At the same time, mutations that arise in the copies in the course of evolution push toward evolutionary divergence of the copies and thus toward the cessation of duplication/deletion (because homology is required for tandem duplication/deletion), while gene conversion events push to make the copies the same again, a situation where copies are more likely to disappear. Evolution here is a reversible process where the long-term outcome depends on a balance of forces. Note also that, in this case, gene conversion may be seen as simplification, and diversification as complexification, and that the opposing tendencies to duplicate and specialize on the one hand and to equalize and collapse on the other may be part of maintaining a balance between over-specialization (or “over-fitting”) and over-simplification, showing the importance of the “ecology of information” for evolution.

As another example, consider the evolution of CpG content, which plays a role in gene regulation and therefore development (Suzuki and Bird 2008; Deaton and Bird 2011). The cytosine in CpG dinucleotides mutates into thymine at a high rate after it is methylated (Hodgkinson and Eyre-Walker 2011), causing CpG-poor islands to lose their CpGs (Mendelson-Cohen et al. 2011). Importantly, this cytosine is methylated by complex enzymatic processes (Klose and Bird 2006), which means that the locations of these mutations are determined by these biological processes (Livnat 2013). At the same time, another mutational force—that of biased gene conversion—adds cytosine to some CpG-poor islands (Galtier et al. 2001; Duret and Galtier 2009), and the balance of such forces determines the direction of the evolution of CpG content (Mendelson-Cohen et al. 2011). The fact that this balance affects in the long term functional, adaptive structure (Suzuki and Bird 2008; Deaton and Bird 2011) fits with interaction-based evolution (Livnat 2013) but is perplexing from a traditional perspective.^29^ Note that CpG mutations have been estimated to account for nearly 25% of all point mutations in humans^30^ (Fryxell and Moon 2005).

As yet another example, based on an analysis of short open reading frames in yeast, Carvunis et al. (2012) have suggested that the evolution of new genes is gradual and reversible: that a new gene does not arise suddenly as a complete whole, but gradually through forms more and more resembling a complete gene; and that at each point in time, the gene can make a step toward or away from completion. I argue that this process too is driven by a balance of forces. In mammals, for example, it would involve the evolution of CpG content discussed above, among other things.

Finally, consider the proliferation vs. silencing and removal of transposable elements (TEs). The divide between those who think that TEs are serviceable to the organism (e.g., McClintock 1965; Britten and Davidson 1969; Lynch et al. 2011; Shapiro 2011; Fedoroff 2012) and those who see them as “selfish-elements” (Dawkins 1976; Orgel and Crick 1980; Doolittle and Sapienza 1980) is well known. Supporting the former, it is now clear that TEs play a major role in adaptive evolution (e.g., Bourque et al. 2008; Sasaki et al. 2008; Fechotte 2008; Lynch et al. 2011). However, the evolutionary “benefit” they bring resides in a timescale too long to allow them to be interpreted as something other than selfish elements under the traditional view. However, as argued above (Sect. 5.3) the writing of mutations is an ecology; it is not machine-like. TEs may well act as though they are propelled to replicate and insert themselves wherever they can, and yet, in the context of the rules of the evolving information ecology, they may be serving the evolution of the organism in the long-term.^31^ Indeed, giving contra-pressure to TE proliferation is an extensive and phylogenetically deep system of regulation, involving methylation and TE removal, active in the germline (Thomson and Lin 2009). I agree with Fedoroff (2012), that this extensive system does not merely act as an “immune defense,” and furthermore argue that it is actually a part of the mutation-writing ecology.

The four examples above clarify the view of genetic change as an ecology of information. It is a view of conflicting forces pushing against each other, including long-term processes that may be locally reversible. This ecology of information computes in the long-term and involves the evolution of the network as a whole: the network gradually changes as it finds where it can give way under this complex set of forces. Thus, through mutational writing, and under natural selection, the evolving network processes a large amount of information.

### Evolutionary Mutational Mechanisms: A Field Open to Future Study

Interaction-based evolution opens up the search for non-Lamarckian yet useful mutational mechanisms. Earlier I proposed a gene-fusion mechanism that may play a role in evolution reminiscent of the role that Hebbian learning plays in neural networks (Sect. 2; see also Sect. 5.7): copies of genes that are used together are more likely to be fused together. This type of mechanism would not cause accidental changes but rather would produce evolutionarily useful genetic variation, without violating the principle that mutations do not respond to the immediate environment.^32^

Another example is as follows. A gene that is highly expressed and is therefore extensively used may be more likely to be duplicated via reverse transcription or other transcription-coupled mutational mechanisms. This may be useful because such a gene may be needed in yet higher quantities or may have a greater potential to beneficially specialize evolutionarily into different functions. As in the case of the gene fusion mechanism of Sect. 2, this can happen also when the gene is extensively used in the soma, because information about the pattern and extent of expression of a gene is present in the DNA and accessible in the germ cells in principle, and may be manifested by the transcriptional promiscuity of the germline (see Sect. 2).

When operating in unicellulars, such mechanisms could explain, among other things, cases of rapid adaptive evolution in response to environmental pressures such as extreme temperatures, extreme salinity, or toxins, where a gene whose product is in demand is duplicated/amplified (Kondrashov 2012). Indeed, at first glance, they may seem Lamarckian, but they are essentially not so. While in unicellulars, environmental pressures may directly cause the overexpression of a gene and thus increase its propensity to be duplicated through mutational mechanisms, in general it is evolution itself that would lead to a situation where a gene is highly expressed and to the application of the same mutational mechanisms in a non-Lamarckian, yet useful, fashion. The fusion of *CypA* and *Trim5* serves as an example here as well, since it involved duplication of *CypA* through retrotranscription, and extensive transcription of *CypA* in the germline may have facilitated it (Kaessmann et al. 2009).

Also of interest in this regard are cases such as the evolution of insecticide resistance in the mosquito *Culex pipiens* due to the amplification of the genes coding for two non-specific esterases as well as for the acetylcholinesterase that is the main target of the applied insecticides, which is active in the central nervous system (Lenormand et al. 1998; Labbé et al. 2007). These duplications may have originated not by accident but by a gradual albeit rapid process of evolution involving natural selection and non-accidental mutation, which has created the genetic conditions under which the duplication mechanisms are more likely to be activated.^33^

Interaction-based evolution draws our attention to the fact that mechanisms such as the above can exist and puts them front and center. The examples above demonstrate that the evolving organism can receive feedback on which genetic changes would be useful to attempt—for example, which genes may be beneficially chunked together. Furthermore, this feedback comes not from the immediate environment, but from the population’s past successes under natural selection. That is, the information is in the genome; no Lamarckism is required.

By accepting that mutation is not accidental, we open the door to examining future research questions that would not have come to light otherwise. Indeed, we may expect a diversity of mutation-writing mechanisms in nature that interact with each other, and the above are merely two examples of these. While some of these mechanisms may be well known phenomena that have not yet been placed within a theoretical framework—for example the fact that recombinational mechanisms interact with DNA sequences in such manner that enables whole gene duplication and deletion—many others may remain to be discovered, like the innumerable details of the still-living RNA world and how it interacts with evolution (Brosius 2005; Morris and Mattick 2014; Knisbacher and Levanon 2015).

### The Intimate Relationship Between Useful Change and Error Repair

It is so often assumed that mutation is a replication error that one might think that this is a well-known scientific fact. However, the only fact that has actually been established is the basic observation itself—that while some genes are duplicated, others undergo genetic change. To say that these changes represent nothing more than “replication errors” is to provide merely one interpretation to this fact, and it may be a prejudiced one. This interpretation has led, among other things, to the terms “error-repair mechanisms” and “error-prone repair mechanisms,” which, according to the theory presented here, may end up detracting from our understanding of evolution.

Error is often a deviation from a pattern. By noticing a deviation from a pattern we can find and fix a typographical error: a word with a typo slightly differs from many correct instances of that word which are all identical to each other; and it can be fixed by making it identical to those other instances. Then again, by noticing a deviation from a pattern, we can also avoid picking a rotten apple in the store, even if we have never seen rot or an apple before. Taking one step further, by noticing a deviation from a pattern we can also spot an error of thought. Take for example *Scala Naturae*, according to which all organisms fall into a linear order from the simplest to the most advanced. From that perspective, the fact that many organisms are hard to classify as more or less advanced in relation to each other is a deviation from a pattern. By replacing *Scala Naturae* with Darwin’s concept of common descent, this difficulty of classification becomes not a deviation from a pattern but a part of a larger pattern involving other facts. Thus, both errors of typing and errors of thought can be corrected by pattern completion at different levels.

At the same time, *pattern completion is a form of simplification:* as information theory makes clear (Li and Vitányi 1997), the fewer exceptions to a rule we need to have, the smaller the amount of information needed to describe the entire system and its parts. Thus, if pattern-completion operations can be implemented by mutation, we may see the same genetic mechanisms operating both in “typographical corrections” and in the kind of mutational writing that leads to progressive evolution. As an example, in the case of duplication and deletion discussed in Sect. 5.4, repeated events of gene conversion have the potential to correct “typos,” but they also have the potential to implement simplification pressure opposing the complexification pressure of diversification.

The insufficiency of our current jargon is made particularly clear by the phrase “error-prone repair.” Suppose that, in some cases where so-called “error-prone repair” is activated, the biological system is actually pushing for a change rather than a restoration of the genetic state, and that this change is a part of a pattern-completion operation or other progressive evolution of the network as a whole. Then we are looking at a mechanism that is not prone to typographical errors, but that is correcting errors in the construction of a fit organism. In other words, what we have heretofore thought of as “error-prone” is actually an attempt at “error-correction,” where the “error” is of a different, deeper kind than we are accustomed to thinking about.

### Evolution as Learning

From Paley (1802) to Dawkins (1986), there is universal agreement that adaptations are incredibly impressive and complex pieces of “natural technology.” While Paley used this observation to make the non-scientific point that, much like a watch has an intelligent watchmaker, life was created by a supernatural intelligence (Paley 1802), Dawkins argued that the process responsible for life is a very simple process of accidental mutation and natural selection that is fully understood at its essence. In the preface to *The Blind Watchmaker* (Dawkins 1986), he wrote that “[t]his book is written in the conviction that our own existence once presented the greatest of all mysteries, but that it is a mystery no longer... Darwin and Wallace solved it, though we shall continue to add footnotes to their solution for a while yet.”

Let us now revisit this question, but from a strictly scientific perspective, and without assuming that all that is essentially important was already revealed at Darwin’s time: Could the process generating life forms and the process generating artificial technology be similar in some respects?

Interestingly, according to Papaj (1993), it is a curious historical fact that the earliest ideas on evolution, i.e., Lamarckism, revolved around observations on *automatism in behavior:* observations showing *that instinct is similar to well-learned behavior*—an evolved phenomenon is similar to a learned phenomenon—in that both can be carried out automatically and independently of external influences, and both are stereotyped, or robotic, repeating with a high degree of uniformity. These observations fostered the idea that what is repeated many times over the generations gradually impresses itself upon the hereditary makeup of the organism, which led in turn to the additional but erroneous idea of Lamarckian transmissionism. Until now, Lamarckism has been the only alternative to natural selection at the most basic level of analysis. And even though it has been rightfully rejected as a general-level explanation for evolution, the observations it was supposed to explain are still here (and have been discussed in Sect. 4). That is, the controversy was never over the *observations* but rather over the *mechanism* of evolution. The current paper provides a new interpretation of these original observations and suggests that there *is* a connection between evolution and learning: network-level evolution and automatization are key to both. This connection is free of Lamarckian transmissionism and requires a process based on non-accidental mutation and natural selection.

Not only in evolution, but also in the study of brain and behavior, the notion of random generation and filtering was once used. For instance, Skinner had suggested a mechanism of random generation of ideas and filtering for how learning by the brain works (see Fodor and Piattelli-Palmarini 2011). However, more recently, Fodor and Piattelli-Palmarini (2011) argued that such a mechanism applies neither to evolution nor to the brain.^34^ In this paper and earlier (Livnat 2013), I argued that the mechanism of evolution is not that of random generation and filtering, and that the causes of mutation are critical for our understanding of evolution. This may also inform our understanding of learning.

Recently, Valiant (2009, 2013) attempted to connect evolution and *machine* learning (see also Feldman 2008, 2009a, b; Kanade, 2011 and Angelino and Kanade 2014 in the same line; and Chastain et al., 2014). Methodologically, his work signifies a turning point: unlike classical population genetics, it provides rigorous mathematical techniques that capture analytically a complex phenotypic structure and allow us to quantify and study the evolution of complexity (Valiant 2009, 2013). Thus, with respect to theoretical methodology, it is a grand vision and, in principle, it allows mutation to be non-accidental. However, while Valiant’s framework allows for, but has not yet substantially pursued, non-accidental mutation,^35^ interaction-based evolution argues that mutation *is* non-accidental and that this is crucial for evolution (Livnat 2013). And while Valiant’s work may be an inspiring step in the right direction, according to the present paper, there are elements that are not yet included in it that are essential for biological evolution based on non-accidental mutation. These include cooption (Sects. 2, 4); the idea that simplification under performance pressure produces elements that have the inherent capacity to become useful in new contexts, which leads to cooption (Sect. 3); the idea that learning through evolutionary change is a learning from the environment by emancipation and acceleration (Sect. 4.14), i.e., by the evolution of automatization and innateness; and the concept of the absorption of meaning from context under gradual network-level change. Indeed, the importance of cooption in evolution cannot be overestimated, and has been demonstrated here at both the molecular and organismal levels (Sects. 2, 4). Furthermore, cooption is analogous to an analogy or metaphor, which are crucial in the evolution of language (Deutscher 2010) as well as in human intelligence. It may be of much interest to explore these missing elements from a computational perspective.

Since its inception (Livnat 2013), interaction-based evolution has been deeply connected to the computational worldview (Papadimitriou 2007; Karp 2011), because it proposed that mutation is an event of information flow and computation: the inputs into a mutational event are the alleles at the loci affecting the mutation through genetic interaction, and the output is the mutation itself (by “mutation” I mean not only a change in the DNA but any heritable change, such as an epigenetic change) (Livnat 2013). Furthermore, the fact that the output of a mutational event at one generation, namely the mutation itself, can serve as an input into mutational events at later generations means that the mutation-writing phenotype creates a network of information flow through the generations, from many genes into any one gene and from any one gene to many (see Fig. 3) (Livnat 2013). Other examples of networks of information flow and computation include the brain, and what computer scientists call a circuit (Papadimitriou 2003; Wegener 1987) (one instance of which is an artificial neural network; Hopfield 1982). Thus, according to interaction-based evolution, genetic evolution can be seen as the result of the workings of a network, itself evolving over time.

Interestingly, in artificial neural networks, local computational elements are used such as Hebbian learning (e.g., Hopfield 1982). In the latter, when one neuron persistently participates in causing another to fire, the strength of the synapse between them is increased (Hebb 1949). Hebbian learning is an example of a local simplification operation that, in the context of the gradual change of a complex network, is useful. Now, elements of this sort can play a role in the network of information flow generated by sex and non-accidental mutation proposed by interaction-based evolution (Livnat 2013); indeed, the mutational fusion mechanism in Sect. 2 is one such case. Thus, we see in multiple ways that, according to interaction-based evolution, evolution and “thinking processes” have more to do with each other than previously thought, even though no Lamarckism and no “foresight” or “adaptive mutation” as traditionally defined are involved. Thus, evolution may be seen as a learning process, and the study of evolution could inform the study of learning and vice-versa.

Recently, a connection between evolution and learning was drawn by Watson and Szathmáry (2016) and Watson et al. (2016). While this connection shares with the theory of interaction-based evolution as proposed earlier (Livnat 2013) and here the idea that evolution is network-based, and that the change of connections between the nodes of the network is key, there are also some fundamental differences between the two. Watson and Szathmáry (2016) and Watson et al. (2016) did not argue for non-accidental mutation, and all that follows from it.

For example, it follows from non-accidental mutation that Hebbian learning–like mechanisms can be implemented directly by the mutational mechanisms themselves (as opposed to needing to arise from accidental mutation and natural selection; Watson and Szathmáry 2016), as discussed in Sect. 2. There, I argued that genes that are used together are fused together.^36^ More generally, I argued that simplification can be implemented by mutational mechanisms. In fact, the very concept of non-accidental mutation itself represents a vast network, as discussed here and by Livnat (2013). Conceptualizing mutation itself as the outcome of network-based processes provides a far more involved network-based view of evolution than otherwise, and greatly strengthens the connection between computer science and evolution (Papadimitriou 2007; Karp 2011).

In addition, borrowing from knowledge in machine learning, the above-mentioned authors mention that, among other things, imposing parsimony pressure by imposing a connection cost in models of genotype-phenotype maps can facilitate evolution in these models (Watson and Szathmáry 2016; Watson et al. 2016; Clune et al. 2013; Kouvaris et al. 2015). However, they do not put simplification pressure front and center in biological evolution, as done here. The present paper established the importance of simplification in biological evolution by providing both the rationale and many empirical examples from both the molecular and organismal levels behind this point. On this foundation, it argued that biological evolution is driven by two forces—the pressure for performance and the pressure for simplification. A cycle in evolution begins at a fuzzy state from the emergent interactions between preexisting elements. From these interactions, simplification under performance pressure creates new elements that have the inherent capacity to come together into unexpected, useful interactions with other such elements. This leads to cooption, and to the beginning of another cycle in the process. Thus, putting simplification front and center in biological evolution also puts cooption at the heart of the evolutionary process. In addition, from this cycle we also obtain the idea that simplification leads to biological complexity (Sect. 3). Indeed, simplification, according to interaction-based evolution, is a creative force.

Following the logic of Clune et al., one would need to justify biologically a substantial cost to a single genetic connection per se if traditional selection is to simplify a genetic network based on random mutation (Clune et al. 2013). In contrast, interaction-based evolution argues that simplification is an active, mechanistic force in biological evolution, and that it can be implemented directly by mutational mechanisms. Indeed, this paper has argued that many empirical observations, from gene translocation and fusion (Sect. 2), gene conversion and deletion (Sect. 5.4), and pattern completion and error-repair (Sect. 5.6) on the genetic level, to the removal of rudimentary organs, the evolution of innateness, the evolution of stereotypy and ritualization, the evolution of an adaptation from fuzzy to sharp and more on the organismal level are better explained by active simplification under performance pressure than by minute costs and benefits under accidental mutation and natural selection. Furthermore, interaction-based evolution argues that mutational mechanisms, mixing of the hereditary material (which has evolved into sex), simplification and selection have all existed from the “beginning” of life.^37^ They did not evolve from an asexual world with accidental mutation, since that world never existed (Livnat 2013). Thus, they are not elements that evolved by accidental mutation and natural selection based on different costs and benefits imposed by that process, but rather are primary elements that are original and inherent to the process of evolution. Thus, interaction-based evolution is different from the evolvability view present in previous biological literature (see more in Sect. 5.8).

Indeed, interaction-based evolution provides a complete, biologically motivated, conceptual framework for evolution with non-accidental mutation at its center. By arguing that novelty arises from emergent interactions, it places the source of novelty at the system level. This in turn replaces the notion of accidental mutation as the ultimate source of heritable novelty, which in turn connects back to the center-piece of non-accidental mutation. This entire framework and all of its elements, including cooption, novelty and non-accidental mutation, as well as the idea that simplification leads to complexity, and the idea that evolutionary learning occurs through automatization and innateness, have not been discussed in previous papers on evolution and learning.

### How Interaction-Based Evolution differs from Previous Theories Involving Non-random Mutation

Lamarckism and notions of directed or adaptive mutation that it influenced imply that an organism can respond beneficially through genetic change to the immediate environment. Thus, if an organism is moved from environment *A* to environment *B*, its mutational tendencies may change accordingly (Futuyma 2009, p. 282). In contrast, according to interaction-based evolution, the probabilities of genetic change are affected by information that is in the genome and that has been the outcome of past evolution over the generations under the joint action of selection and continually evolving mutational tendencies—these probabilities are generally not affected directly by the environment (see more in Sect. 5.5). Thus, when an organism is moved from environment *A* to *B*, its mutational tendencies may well remain the same during its lifetime, even though they are non-accidental and still respond to past, long-term selection pressures.

Interaction-based evolution shares with past works on evolvability and on modifiers of the mutation rate (Kimura 1967; Leigh 1970; Sterelny 2007; Koonin 2011; Ram and Hadany 2012, 2014) the basic recognition that there are evolved genetic influences on mutation. However, the latter have assumed either implicitly or explicitly that accidental mutation is at the core of the adaptive evolutionary process, first by relying on accidental mutation and natural selection for the evolution of mutational mechanisms in the first place, and second by treating these mechanisms as helpful but not essential add-ons to the core process of accidental mutation and natural selection. In contrast, interaction-based evolution argues that biological evolution has always depended essentially on the joint action of mutational mechanisms and natural selection, and indeed, none of its main points have been proposed by past literature on evolvability (see more in Sect. 5.7).

Furthermore, evolvability sensu mutational mechanisms only allows a repetitive pressure to lead to a generic mutational response to that pressure. For example, the supposed S.O.S. system in bacteria is thought to have evolved to increase the *general accidental* mutation rate as a generic response to starvation (Radman 1999; Caporale 2003; Koonin 2011). In contrast, I argue that an ever-present force of mutational mechanisms is part of evolution in general, because evolution is always a joint evolution of a mutation-writing phenotype and a performing phenotype (Livnat 2013). Therefore, continually evolving, per-locus probabilities of mutation affect the evolution of each adaptation specifically in an ongoing manner even if the pressure for it is not repetitive but appears only during one evolutionary period of time. For example, in the discussion of the *TRIM5-CypA* fusion in Sect. 2, I proposed that adaptive evolution in response to pathogen pressure, which had made these two genes come to work tightly together, had affected the fusion probability of these two particular genes, which has driven the evolution of this adaptation further. Indeed, related to this difference between interaction-based evolution and evolvability, only interaction-based evolution raised the clear prediction that there is a relatedness in mutational tendencies—that mutations are more similar the more closely related the entities under consideration are (Livnat 2013)—and that these tendencies are of fundamental importance for adaptive evolution (Livnat 2013).

Among those who have discussed non-accidental mutation, Shapiro and Caporale deserve much credit for promulgating this idea (Shapiro 2011; Caporale 2003). Shapiro sees mutation as “natural genetic engineering” (Shapiro 2011) and has made an analogy between mutation (especially, transposable elements activity) and the formatting of a computer drive (Shapiro 2002, 2011). Caporale has also examined examples of non-accidental mutation at the molecular level (Caporale 2003). While I share with both the basic recognition that mutation is non-accidental, and with Shapiro the belief in the importance of the computational view, none of the main ideas of interaction-based evolution are present in their work.

Finally, it is instructive to consider orthogenesis of the nineteenth century (Gould 2002) as a theory of non-random mutation. However, orthogenesis and related ideas have been proposed either as a complete replacement to or as an auxiliary force under natural selection (Gould 2002). Interaction-based evolution argues instead that internal factors, i.e., mutational mechanisms, and natural selection (in the sense of differential survival and reproduction) work jointly as two complementary forces—simplification under performance pressure—to drive adaptive evolution.

## Conclusions

How does novelty arise? Traditional evolutionary thinking relies on accidental mutation and natural selection. The idea is that radiation, replication errors, oxidative stress, etc. cause local genetic accidents that, on rare occasions, provide beneficial phenotypic changes. All that remains for natural selection to do is to check whether a mutation is beneficial or detrimental—to play the role of a sieve. Where does the new genetic information come from? Presumably, in that view, it comes from the accident itself, and there is nothing more to inquire about the source of it that is essential for our understanding of the process. As explained, one important amendment to this view has been evolvability. However, evolvability sensu the genotype-phenotype map focuses on the translation of genetic information to phenotypic change, not on the causes of genetic change; and evolvability sensu mutational mechanisms still relies, implicitly or explicitly, on accidental mutation at the core of the process. Another important approach has been that of the modern literature on novelty; but while this approach is consistent with the present paper in multiple important ways, it does not focus on the question of the causes of mutation.

Interaction-based evolution proposes an alternative. The mutations that are relevant for adaptive evolution under selection are due to mutational mechanisms that are continually evolving, and that do not in and of themselves invent things. Rather, novelty arises from the system level—from the macroscale—from gradual network-level evolution, as these mechanisms absorb information from selection. In brief, mutational mechanisms perform simplification operations on the genetic network, as well as gene duplication, in a heritable mode. These mechanisms work together with natural selection (which acts on the organism as a complex whole, not on single genes as independent actors), so that adaptive evolution is a process of simplification under performance pressure. A cycle in this process begins with complex, high-level interactions between preexisting elements. Simplification under performance pressure takes these preexisting interactions and, gradually, over the course of evolution, creates from them new elements and new adaptations. Because these new elements are created in a process of simplification under performance pressure, they have the inherent capacity of coming together in new, useful and unexpected interactions at higher levels, thus initiating another cycle in the process. This capacity to come together in useful high-level interactions that have not been pursued in advance is the source of novelty in evolution. In short, mutations do not in and of themselves invent things, but rather are a key activity that takes part in turning the wheel of evolution. Furthermore, it is simplification that explains complexity: local simplification leads to a global increase in complexity.

Thus, while traditional theory is based on the idea that accidental mutation invents—where this supposed accidental mutation is a remote, presumed event that cannot be seen or confirmed—the theory presented here is based on the empirically evident fact that preexisting elements come together into new, useful high-level interactions as the source of novelty in evolution. Note that it matters not whether the novelty involved in the transitions from the genes *TRIM5* and *CypA* to their fusion, or from haphazard egg retrieval to backward walking, is small or great in and of itself. Rather, these transitions exemplify the steps that tie together the process of evolution, which in the long term has generated eyes, brains, etc.

We have a tendency to look for “foundations” from which everything else can be derived. In particular, it is convenient to assume that the causes of mutation are random, because it puts an end to all of our questions. The philosophical move that is required from the perspective of interaction-based evolution is to let go of the notion that random mutation and novelty from a point are at the bottom of things—that they provide a stable ground upward from which a conceptual edifice can be built; and to accept instead that the action is at the network level: that both the meaning *and origin* of genetic and phenotypic elements comes from the higher levels of organization—it comes from the network—from “above.” This move opens up the study of evolution substantially; because while the notion of random mutation means that there is nothing of importance to be studied about the causes of mutation from an evolutionary perspective, the concept of non-accidental mutation provided by interaction-based evolution implies instead a whole world of biological mechanisms open to investigation.

Before Darwin, people used to think that different species were each created separately in an instant. While Darwin made an immense contribution by showing that this is not the case, and that species are generated gradually, a notion of creation in an instant has been maintained in neo-Darwinism in other areas: the origin of life, the origin of mutations, and cooption. While Livnat (2013) argued among other things against the origin of life in an instant, this paper argues against the other two. Novelty arises not suddenly from a point but from gradual network-level evolution. Indeed, if evolution according to random mutation and natural selection is a sequence of independent points, each representing a local accidental mutation disconnected from the rest, interaction-based evolution draws the lines between these points (see Livnat 2013) while fundamentally altering their interpretation.

## Footnotes

1 This sense of evolvability is distinct from that of Valiant (2009), to be discussed in Sect. 5.7.

2 At most it can be used in addition to accidental mutation and natural selection, but that does not bring us closer to a parsimonious and general-level explanation for the remaining mysteries of evolution. Already in 1926, Berg commented on the lack of parsimony in using both a selection-based mechanism and an independent, Lamarckian-based mechanism to explain different cases of adaptive evolution (Berg 1926).

3 Note that here I mean “natural selection” in the sense of differential survival and reproduction, not in the sense of the traditionally proposed process consisting of accidental mutation and selection as a sieve.

4 It has been part and parcel of the rise of evo-devo, a field unfortunately neglected at the time of the modern synthesis (Müller 2007a).

5 Many organismal-level observations will be provided in this paper and their role is to support the fact that, also at the organismal scale, evolution is based on network-level evolution and simplification under performance pressure. These in turn connect with non-accidental mutation in fundamental ways, as will be explained in Sect. 5.2.

6 As will be discussed in Sect. 5.7, a connection between Hebbian learning and evolution was recently proposed via phenotypic integration (Watson and Szathmáry 2016; Watson et al. 2016). In contrast, here it is proposed that a Hebbian learning–like mechanism is implemented by mutation itself.

7 This to-and-fro movement is not surprising, as it is very common in territorial disputes across species of birds, fish and mammals.

8 “Number” of modules and “more” modules could be put in quotations because modules do not have a precise number, as they ultimately grade into each other, indeed because they have to be connected to each other. The definition of a module used in the literature is a fuzzy one and rightly so: it is a set of genes that interact more closely with each other than with other genes, even though to interact with the “outside,” at least some of its members have to have just as strong a connection to members outside of the module. However, the fact that we cannot perfectly count the total number of modules is an inherent characteristic of the process: it allows new modules to gradually form.

9 See Deutscher (2010) and Byers (2010) for conceptually relevant processes in other realms.

10 For modern literature on novelty, see Sect. 4.16.

11 We now know that genetic elements previously thought to participate in “one” gene actually form products together with elements previously thought to belong exclusively to “other” genes, and so the boundaries between genes have been blurred.

12 This term, which aptly describes one of the most important points of this paper, was proposed by Nick Pippenger.

13 While excellent reviews are available on the nineteenth century literature on this very general question (Gould 1977, 2002), I do not know of substantially different modern ideas besides the informal use of economical considerations.

14 Remarks: First, the work referred to requires no Lamarckian knowledge of the macroscale phenotype and the immediate environment, but genetic influences on mutation can still evolve in the long-term based on the principles of interaction-based evolution. Second, while one may argue that simplification under performance pressure could work in addition to rather than instead of accidental mutation and natural selection, the goal of this paper is first and foremost to provide a conceptual alternative to accidental mutation and natural selection as a sieve, rather than to decide whether or not accidental mutation is in operation as far as adaptive evolution is concerned. Third, for more on the connection between simplification and non-accidental mutation, see in Sect. 5.2.

15 The pattern consisted of an absence of or a gap in the posterior crossvein and sometimes the anterior one too.

16 In order to observe this, the experimenters took at each generation a certain sample of flies and raised them without heat shock.

17 In fact, once we assume that Stern’s model taken at face value is alone the relevant mode of explanation, it would have been easier to assume that complex instincts in nature evolve afresh, without relation to a preexisting behavior modulated by a brain and modified by the environment, because the model does not describe a world where such a relation is biologically reasonable—it requires a brain that affects independently each threshold expediently assumed for each particular trait that is to become innate, each with its own expediently assumed set of additive alleles.

18 But see the lively debate in the nineteenth century on it (Gould 1977).

19 “Backing” refers to the fact that these dogs copy the posture of another dog that is on point; even this behavior, which is of use to the hunters, is strongly innate.

20 As an example of the animals’ acute discriminatory abilities, a herring gull can recognize its mate among a group of other gulls from 30 yards away (Tinbergen 1951).

21 Note that the argument is that of implication: *If* selection on transient, complex combinations of alleles across loci is at the core of the adaptive evolutionary process, *then* stabilization must accompany adaptation. This does not tell us the details of how stabilization happens, which interaction-based evolution argues relate to non-accidental mutations and innateness as discussed in Sects. 2 and 2.3. While Siegal and Bergman (2002) offered a related view, their view does not consider the nature of mutation.

22 Accordingly, when Barlow noted that even what were previously called FAPs are not uniformly fixed, but rather some FAPs vary more than others (Barlow 1977), he tried to explain this continuum by arguing that signals that need to be clearer are more stereotyped, and others are less so (Barlow 1977).

23 This is not to say that all traits must inevitably cover the whole spectrum and reach the extreme, nor that they all move along the spectrum at the same rate; but it is to say that the degree of fixedness comes from the nature of the process of network-based evolution, and is not an independent element traditionally selected for, as Maynard-Smith and Harper (2004) and Mayr (1974) have argued. Indeed, whether the evolving trait is a signal or not, the evolutionary process is converging on an adaptation: along with the decrease in variance and disorder, it converges on highly efficient structure and behavior. Clarity, which is part of the effectiveness of the signal, and efficiency in other adaptations, are outcomes of the evolutionary process, and uniformity is an inherent concomitant of the process in both cases.

24 “Environment” and “experience” need to be qualified: No behavior is entirely independent from the “environment” or from “experience” when these notions are so broadly construed as to include such factors as the flow of food materials during development or the “source of experience” that one body tissue provides a neighboring one in the course of development. Even what we more normally call “experience,” that is interaction with the outside during growth and learning, can figure into and change aspects of behaviors that are otherwise innate. For example, the tendency of the rat to build a nest is innate, yet it will not be able to carry it out if it is deprived of experiencing the carrying and manipulating of objects during development (Lehrman 1953). Despite this important qualification, the conceptualization of innateness as independence from the environment is still true and useful in an important sense.

25 First, both Tinbergen and Lorenz considered it of great importance that stereotyped behavior (fixed action pattern; point 2 above) is also homologizable between species and therefore is as useful for taxonomy as morphological characters are (point 3) (Tinbergen 1951; Lorenz 1981) (Lorenz called it “an epoch making discovery;” Lorenz 1981, p. 103). Second, there is an agreement that, empirically, what is innate in the first sense above (1)—what is automatic—appears also to be stereotyped, or “fixed;” it appears “robotic” (2) (Barlow 1977; Papaj 1993). Indeed, Barlow’s continuum of FAPs runs both from variable to stereotyped and from dependent on to independent of the environment at the same time (Barlow 1977).

26 Of course, these cycles do not occur in a sequence one at a time. At any time point in the course of the evolution of a given life form we may expect many co-occurring cycles, each at a different phase.

27
Darwin (1859) recognized this point, but it has not been sufficiently emphasized during the modern synthesis. As multiple authors have argued (Gould and Vrba 1982; Müller and Newman 2005; Wagner and Lynch 2010), the latter focused on the modification of an existing character under natural selection. The prominent position in twentieth century evolutionary theory of Wright’s shifting balance theory (Wright 1931, 1932), which attempts to explain the evolution of new, beneficial genetic interactions by crossing fitness landscape valleys with the help of random genetic drift, serves as evidence for the neglect of cooption during that period of time. As Hallgrímsson et al. (2012) pointed out, within Wright’s analogy, cooption is like a bridge over a valley from one adaptive peak to another, and does not require loss of fitness.

28 We are no longer restricted to the effective transmission of additive genetic effects.

29 Indeed, as Duret and Galtier argued, CpGs work en masse, and the impact of any one particular CpG mutation is insignificant and could not be explained by traditional natural selection (Duret and Galtier 2009).

30 This is the case even though CpG dinucleotides account for only about 1% of the human genome. Furthermore, they are often accompanied by mutations in nearby bases (Qu et al. 2012; Walser et al. 2008), further compounding the involvement of mutational mechanisms in their origination.

31 This is the case even if they have the appearance of “selfish agents” that has been attributed to them, and even if they occasionally cause accidents in the short-term in the form of genetic disease (Livnat 2013). Livnat and Pippenger (2006) explored from a mathematical perspective in another realm the idea that a system with its own objective function can be composed of conflicting agents.

32 Interestingly, while the mutational fusion mechanism hypothesized earlier is based on putting together empirical observations (namely the nature of transcription, chromatin states, reverse transcription and gene fusion), its general nature was predicted based on the theoretical considerations of interaction-based evolution (Livnat 2013): in the latter, the conceptual connection between the problem of sexual recombination and mutational mechanisms required that genes play a dual role: one of performance under natural selection, and another of influencing mutation in the germline (Livnat 2013). Thus, the coupling of germline mutation and somatic performance through transcription as in the mechanism hypothesized earlier allows for a convergence of diverse empirical and theoretical considerations.

33 Such a process would enable selection on multiple or many loci to be funneled by mutational mechanisms over the generations to influence the probability of duplication of a particular gene or genes.

34 Likewise, Lakatosh (1976) asked whether there is nothing more to intelligence than randomization as generating ideas and selection sifting among them.

35 Valiant’s framework allows mutation to be an outcome of any efficient randomized algorithm. Such algorithms use random bits *in a controlled fashion*, which is different from mutations that are nothing but random changes anywhere in the genome. At the same time, his foundational model (Valiant 2009) attempts to retain an important part of the spirit of accidental mutation in traditional evolutionary biology.

36 In contrast, Watson et al. argue that “genes that are selected together are wired together.” There is a fundamental difference between the two statements, because Watson et al. imply a process of random mutation and natural selection. Namely, they base their statement on a pioneering theoretical model (Pavlicev et al. 2011), but one that is constructed within the random-mutation view.

37 This may not have been a “beginning” but a gradual transition (Livnat 2013).
